# *Trichoderma reesei* XYR1 activates cellulase gene expression via interaction with the Mediator subunit TrGAL11 to recruit RNA polymerase II

**DOI:** 10.1371/journal.pgen.1008979

**Published:** 2020-09-02

**Authors:** Fanglin Zheng, Yanli Cao, Renfei Yang, Lei Wang, Xinxing Lv, Weixin Zhang, Xiangfeng Meng, Weifeng Liu

**Affiliations:** State Key Laboratory of Microbial Technology, Microbial Technology Institute, Shandong University, Qingdao, Shandong, People’s Republic of China; University College Dublin, IRELAND

## Abstract

The ascomycete *Trichoderma reesei* is a highly prolific cellulase producer. While XYR1 (Xylanase regulator 1) has been firmly established to be the master activator of cellulase gene expression in *T*. *reesei*, its precise transcriptional activation mechanism remains poorly understood. In the present study, TrGAL11, a component of the Mediator tail module, was identified as a putative interacting partner of XYR1. Deletion of *Trgal11* markedly impaired the induced expression of most (hemi)cellulase genes, but not that of the major β-glucosidase encoding genes. This differential involvement of TrGAL11 in the full induction of cellulase genes was reflected by the RNA polymerase II (Pol II) recruitment on their core promoters, indicating that TrGAL11 was required for the efficient transcriptional initiation of the majority of cellulase genes. In addition, we found that TrGAL11 recruitment to cellulase gene promoters largely occurred in an XYR1-dependent manner. Although *xyr1* expression was significantly tuned down without TrGAL11, the binding of XYR1 to cellulase gene promoters did not entail TrGAL11. These results indicate that TrGAL11 represents a direct *in vivo* target of XYR1 and may play a critical role in contributing to Mediator and the following RNA Pol II recruitment to ensure the induced cellulase gene expression.

## Author summary

As a model cellulolytic fungus, *T*. *reesei* is capable of rapidly producing a large quantity of (hemi)cellulases when appropriate substrates are present. This outstanding characteristic has made *T*. *reesei* a prominent producer of cellulase in industry and also a model organism for studying eukaryotic gene expression. The expression of these hydrolytic enzymes encoding genes in *T*. *reesei* is precisely regulated at a transcriptional level and controlled by a suite of transcription factors. Among others, the transcription activator XYR1 has been firmly established to be absolutely necessary for activating the expression of almost all cellulase genes. However, the precise mechanism it acts remains largely unknown. In eukaryotes, the multisubunit Mediator complex has been shown to be critical for expression of most, if not all, protein-coding genes by conveying regulatory information to the basal transcription machinery. Here, we find that XYR1 interacts with the Mediator tail module subunit, TrGAL11, which contributes to cellobiohydrolase (*cbh*) and endoglucanase (*eg)* genes but not β-glucosidase (*bgl*) genes expression. Thus, the induced XYR1 binding to cellulase gene promoters led to TrGAL11 and RNA Pol II recruitment to these promoters. These results show that TrGAL11 represents a direct *in vivo* target of XYR1 and provide evidence for not only the evolutionarily conserved function of Mediator, but also for the existence of some subtle difference in its action to mediate gene expression in different eukaryotes.

## Introduction

In eukaryotes, RNA polymerase II (Pol II) transcribes all protein-coding genes whose initiation is dependent on a set of general transcription factors (GTFs), which recognize the core promoter and facilitate transcription initiation from the correct start site. While various additional factors contribute to the regulation of Pol II activity at the promoter, the multisubunit Mediator complex has been shown to be critical for expression of most, if not all, Pol II transcripts [1–3]. The functional activities identified for Mediator thus include influencing preinitiation complex (PIC) formation, stimulating phosphorylation of the Pol II carboxy-terminal domain (CTD) by transcription factor IIH (TFIIH), and interacting with activators and repressors to convey regulatory information to the basal transcription machinery [3, 4].

Mediator is representative of macromolecular complexes made of 21 subunits with a molecular mass of ~0.9 MDa in *Saccharomyces cerevisiae* and 26 subunits (~1.4 MDa) in human [5]. Extensive genetic, biochemical and structural studies have shown that Mediator from both yeast and human is organized into four modules, Head, Middle, Tail that are held together by a backbone subunit MED14, and a more loosely associated Kinase module (CKM) [6]. This distinctive modular structure of Mediator contributes to its multiple layers of function. While the flexibility and extended shape of the head module permit extensive interactions with Pol II as well as with other components of the transcription initiation complex, the middle module confers structural integrity on Mediator and also contacts Pol II [7]. Recent high-resolution structural data have revealed that Mediator undergoes coordinated structural shifts at the extensive interfaces for head-middle module subunits upon binding the Pol II enzyme as well as the activation domains of DNA-binding transcription factors [7–10]. These large rearrangements are considered to allow the structure of Mediator to be geared to access different conformational states required for RNA Pol II interaction and PIC stabilization [7, 8]. The head and middle modules are thus critical for the expression of virtually all protein-coding genes and a majority of subunits are essential for viability [5]. Unlike the fairly rigid head and middle modules, the tail module is relatively flexible, whose main function seems to connect Mediator to sequence-specific transcription factors such as GAL4, GCN4, HSF1 and so on [11–14], as most activator-Mediator interactions described to date involve tail subunits [12, 15–21]. None of the tail module subunits (MED2, MED3, MED5, MED15/GAL11, and MED16) is thus essential for viability [22]. Although most models of yeast Mediator have suggested that it is a monolithic complex, evidence does exist supporting the independent existence of Mediator subcomplexes [23, 24]. In this regard, a tail submodule (MED2/MED3/MED15(GAL11)) can even be recruited independently of the core mediator consisting of the head and middle modules by GCN4 and HSF1 as a free complex [12, 13]. It has been therefore argued that the different forms are involved in regulating different subsets of genes or responding to different groups of regulators (both activators and repressors). Given the potential of the highly dynamic structural complexity for Mediator, and the fact that its subunit composition and sequences have diverged significantly across eukaryotes [25], the functional role of specific Mediator component and the precise mechanism by which Mediator regulates gene expression in different eukaryotic species has yet to be investigated.

The saprophytic filamentous fungus *T*. *reesei* is widely applied in the industry due to its excellent capability of secreting a large quantity of cellulases [26, 27]. In the past several decades, extensive efforts have been made for understanding the intricate regulatory network controlling cellulase gene expression and a suite of transcription factors involved in cellulase gene regulation have thus been identified [28–34]. Among others, Xylanase regulator 1 (XYR1) has been found to be absolutely necessary for activating the expression of almost all cellulases/hemicellulases genes [30, 35, 36]. XYR1 overexpression has been further shown to be able to result in a full expression of cellulases even under non-inducing conditions [37, 38]. Regardless of these, the exact mechanism by which XYR1 activates cellulase gene expression remains largely unresolved.

In this study, we identified a *S*. *cerevisiae* GAL11 homolog, TrGAL11, (jgi:Trire2:107300) in *T*. *reesei* and showed that the KIX domain of TrGAL11 interacts directly with XYR1 activation domain *in vitro*. Deletion of *Trgal11* severely compromised the induced expression of most cellulase genes except that of two major β-glucosidase genes. This differential involvement of TrGAL11 in the expression of cellulase genes was recapitulated by RNA Pol II recruitment on the core promoter of these genes. We further provide evidence that TrGAL11 is recruited to cellulase gene promoters in an XYR1-dependent manner *in vivo*. Nevertheless, XYR1 occupancy on cellulase gene promoters was not affected in the absence of TrGAL11, and elevated XYR1 expression was not able to rescue the defective cellulase gene expression in the *Trgal11* deletion strain.

## Results

### Identification of a *T*. *reesei* GAL11/MED15 homolog that interacts with XYR1

**Fig 1:**
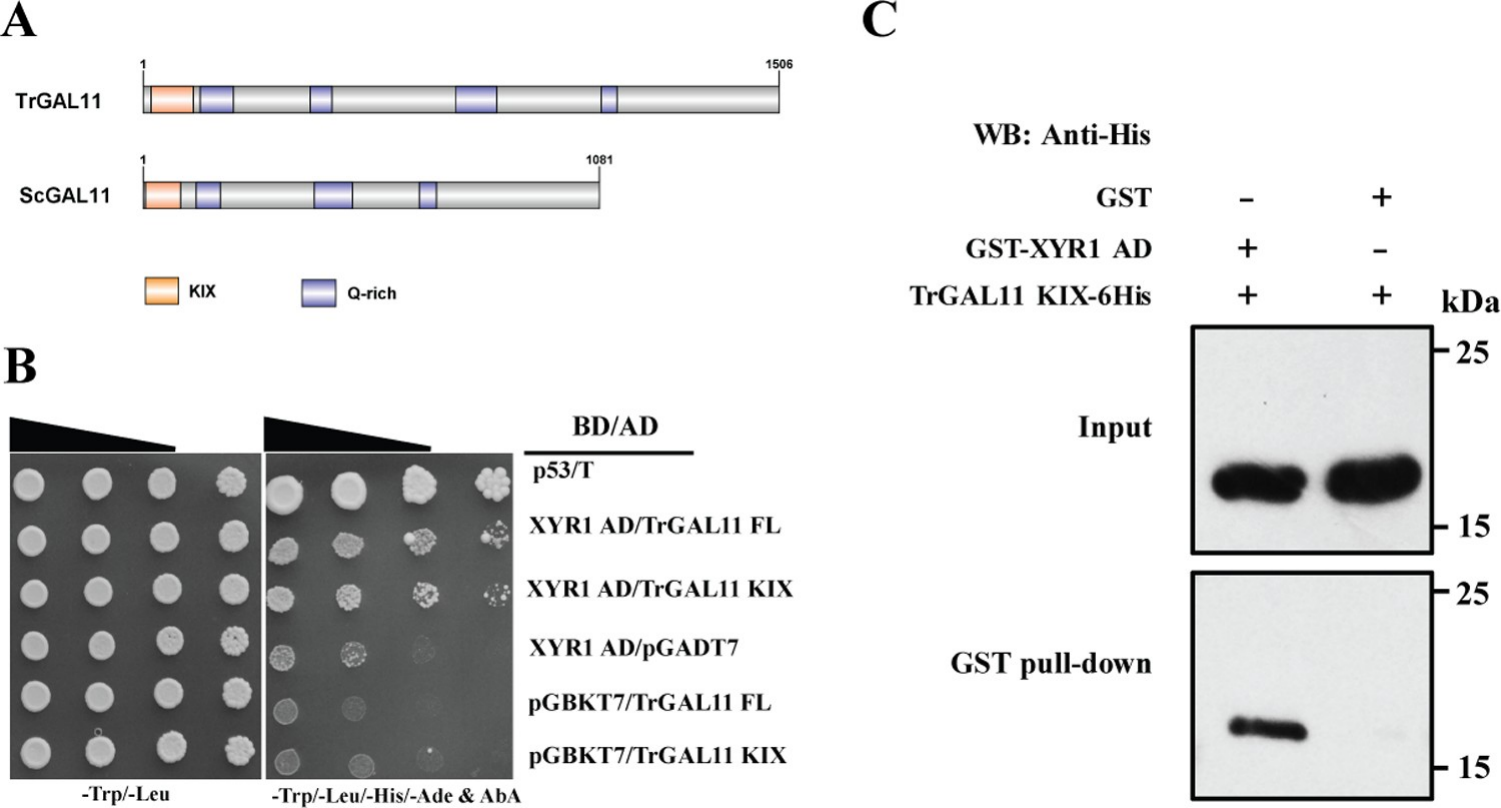
TrGAL11 interacts with XYR1 *in vitro*. (A) Schematic diagram of domain of TrGAL11 and ScGAL11. The KIX domain and glutamine rich region (Q-rich) of each protein are labeled as indicated. (B) Yeast two-hybrid analyses of interactions between XYR1 activation domain (XYR1 AD) and full length or the KIX domain of TrGAL11. Serial dilutions of yeast transformant cells harboring the indicated plasmids were spotted on double dropout medium (DDO, SD/–Leu/–Trp) and quadruple dropout medium (QDO, SD/–Ade/–His/–Leu/–Trp) plates containing 75 ng/mL AbA, respectively, and were allowed to grow at 30°C for 3 days. The p53 plus T was set as a positive control. Significant growth were shown in transformants containing XYR1 AD plus full-length or the KIX domain of TrGAL11 while only very weak growth was observed for control transformants containing pGBKT7 plus pGADT7-TrGAL11 or pGADT7 plus pGBKT7-XYR1 AD. (C) TrGAL11 interacts with XYR1 activation domain (aa 767–860) in a GST pull-down assay *in vitro*. Recombinant TrGAL11 KIX-6His was incubated with glutathione sepharose 4B beads-coupled GST- XYR1 AD_767-860_ or GST as a control. TrGAL11 KIX retained on the beads after extensive washing was detected by western blot with anti-His antibody.

To identify XYR1 coactivators and explore how they may facilitate XYR1-mediated transcriptional activation of cellulase genes in *T*. *reesei*, we focused on the Mediator complex whose tail module subunit GAL11/MED15 has been reported to serve as the target of various transcriptional activators [11, 12, 20, 39, 40]. Direct search in the NCBI protein database retrieved a candidate homolog of ScGAL11 (GenBank: EGR48723.1), which hereafter was named TrGAL11 (jgi: Trire2:107300). TrGAL11 shared a relatively low similarity and was only 23.88% identical with ScGAL11 over the primary amino acid sequence (S1 Fig). However, domain analysis and structural prediction by HHpred [41] and SWISS-MODEL [42] revealed the existence of a conserved N-terminal KIX domain with high structural similarity with those of ScGAL11 (PDB_2k0n) (Figs 1A and S1). Similar *in silico* analyses revealed that the *T*. *reesei* genome also contains predicted orthologs for most other components of the *S*. *cerevisiae* Mediator complex (S1 Table and S2 Fig). Together, these analyses suggest that an evolutionarily conserved Mediator exists in *T*. *reesei* which may be similarly involved in initiating RNA Pol II transcription.

Yeast two-hybrid (Y2H) and GST pull-down assays were then performed to test whether an interaction exists between TrGAL11 and XYR1 (Fig 1B and 1C). While significant growth was observed for yeast transformants expressing the putative activation domain (AD) of XYR1 (767~860 aa) and the full-length TrGAL11 or TrGAL11 KIX domain, only very weak growth was shown for control transformants (Fig 1B). GST pull-down assay further demonstrated that the His-tagged TrGAL11 KIX domain was efficiently retained by the recombinant GST-XYR1 AD but not in the GST coupled beads (Fig 1C). Altogether, these results indicate that XYR1 directly interacts with TrGAL11 *in vitro*.

### TrGAL11 is required for the fully induced expression of (hemi)cellulase genes but not for β-glucosidase genes

**Fig 2:**
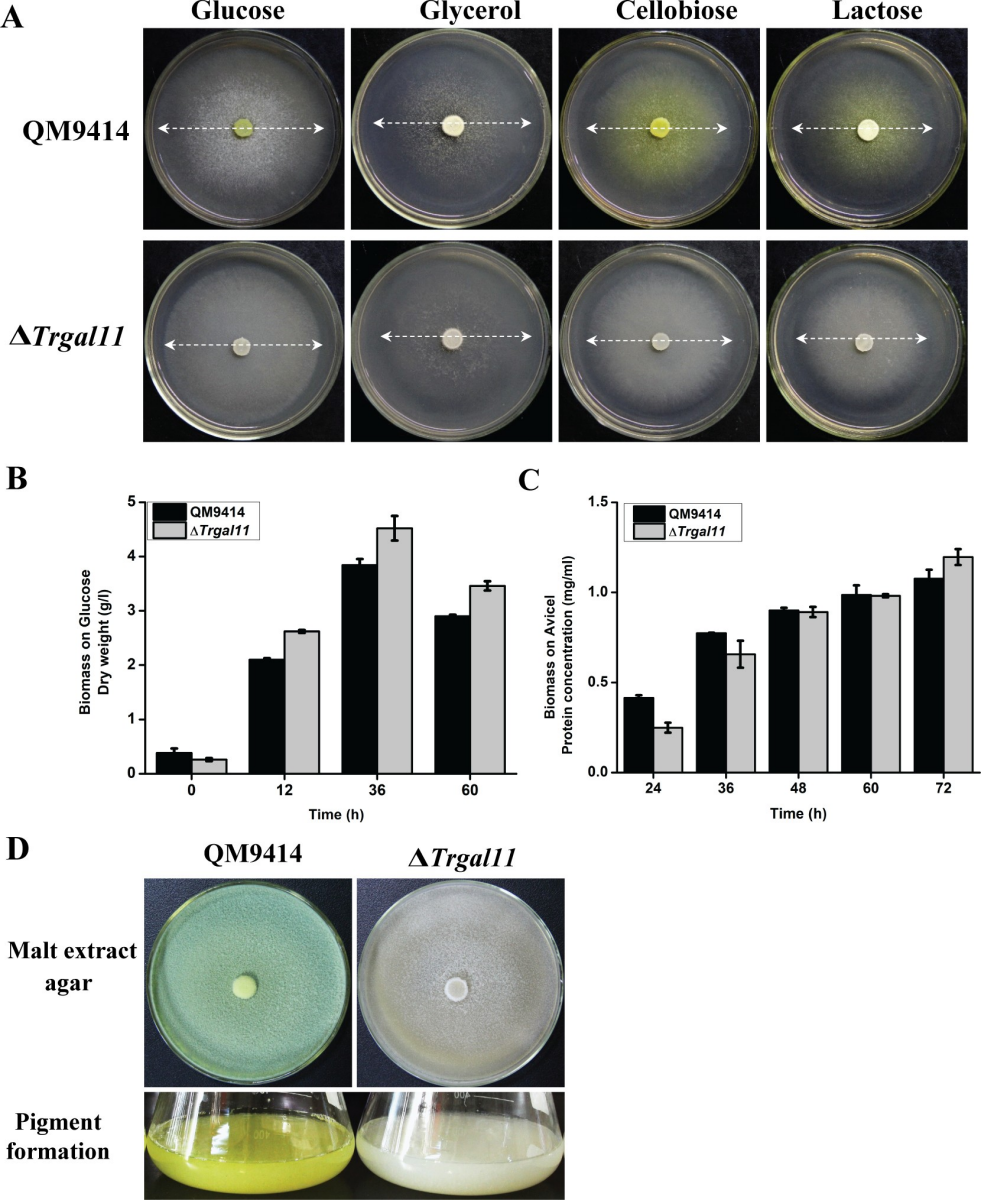
*Trgal11* disruption had little effect on *T*. *reesei* growth on plates or in liquid medium but reduced its conidia and pigment formation. (A) Growth of QM9414 and the Δ*Trgal11* strains on plates with various carbon sources at a final concentration of 1% (w/v) at 30°C for 3 days. (B) Biomass analysis of QM9414 and a representative Δ*Trgal11* in liquid MA medium with 1% glucose as the sole carbon source. The biomass was determined by analyzing the dry weight. (C) Biomass accumulation of QM9414 and Δ*Trgal11* after inoculation of equal amounts of pre-cultured mycelia in liquid MA medium with 1% Avicel as the sole carbon source was determined by analyzing the intracellular protein content. (D) Conidiation and pigment formation analyses of QM9414 and the Δ*Trgal11* strains on malt extract agar. No statistical difference (*t*-test, P>0.05) was observed for the growth of these strains.

To investigate the *in vivo* function of TrGAL11, a *Trgal11* null mutant (Δ*Trgal11*) was generated by replacing the *Trgal11* coding region with the orotidine-5-decarboxylase gene *pyr4* in QM9414Δ*pyr4* strain (S3 Fig). *Trgal11* deletion had hardly any effect on mycelia growth and the final biomass yield in liquid MA medium containing glucose or Avicel as carbon source compared to that of QM9414 (Fig 2A–2C). However, conidiation and pigment formation were compromised in the *Trgal11* deletion strain (Fig 2D). These data indicated that TrGAL11 may play an important role in mediating gene expression involved in asexual reproduction and secondary metabolism in *T*. *reesei*. Interestingly, *Trgal11* disruption resulted in a significantly elevated resistance to hygromycin B and pyrithiamine although the exact mechanism is not clear at present (S4 Fig).

**Fig 3:**
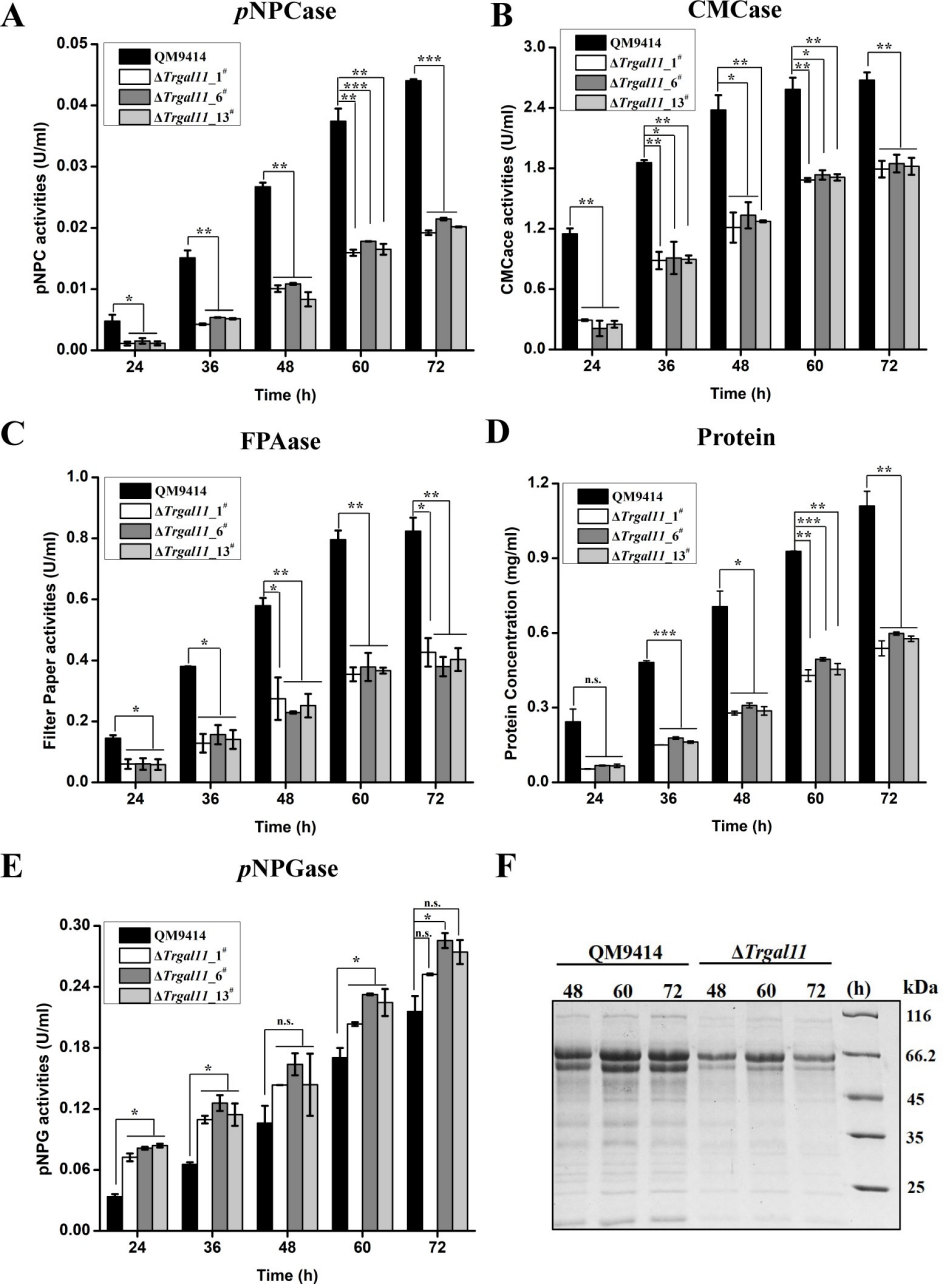
Deletion of *Trgal11* compromised the fully induced production of cellobiohydrolase and endoglucanase but not of β-glucosidase. (A-E): Extracellular *p*NPCase activity (A), CMCase activity (B), filter paper activities (FPAase) (C), protein concentration (D), and *p*NPGase activity (E) of the culture supernatant from the parental strain QM9414 and three independent Δ*Trgal11* transformants cultured on 1% (w/v) Avicel for the indicated time periods. (F) Culture supernatant of QM9414 and the Δ*Trgal11* strains on 1% (w/v) Avicel was analyzed by SDS-PAGE and Coomassie Brilliant Blue staining. As done with biomass quantification in Fig 2C, equal amounts of the same Avicel culture at the indicated time points after inoculation of pre-cultured mycelia were taken for measuring extracellular activity. Significant differences (*t*-test, *P<0.05, **P<0.01, ***P<0.001) were detected for the extracellular activities except *p*NPGase activity between QM9414 and three independent transformants of Δ*Trgal11* for the indicated time points after induction.

**Fig 4:**
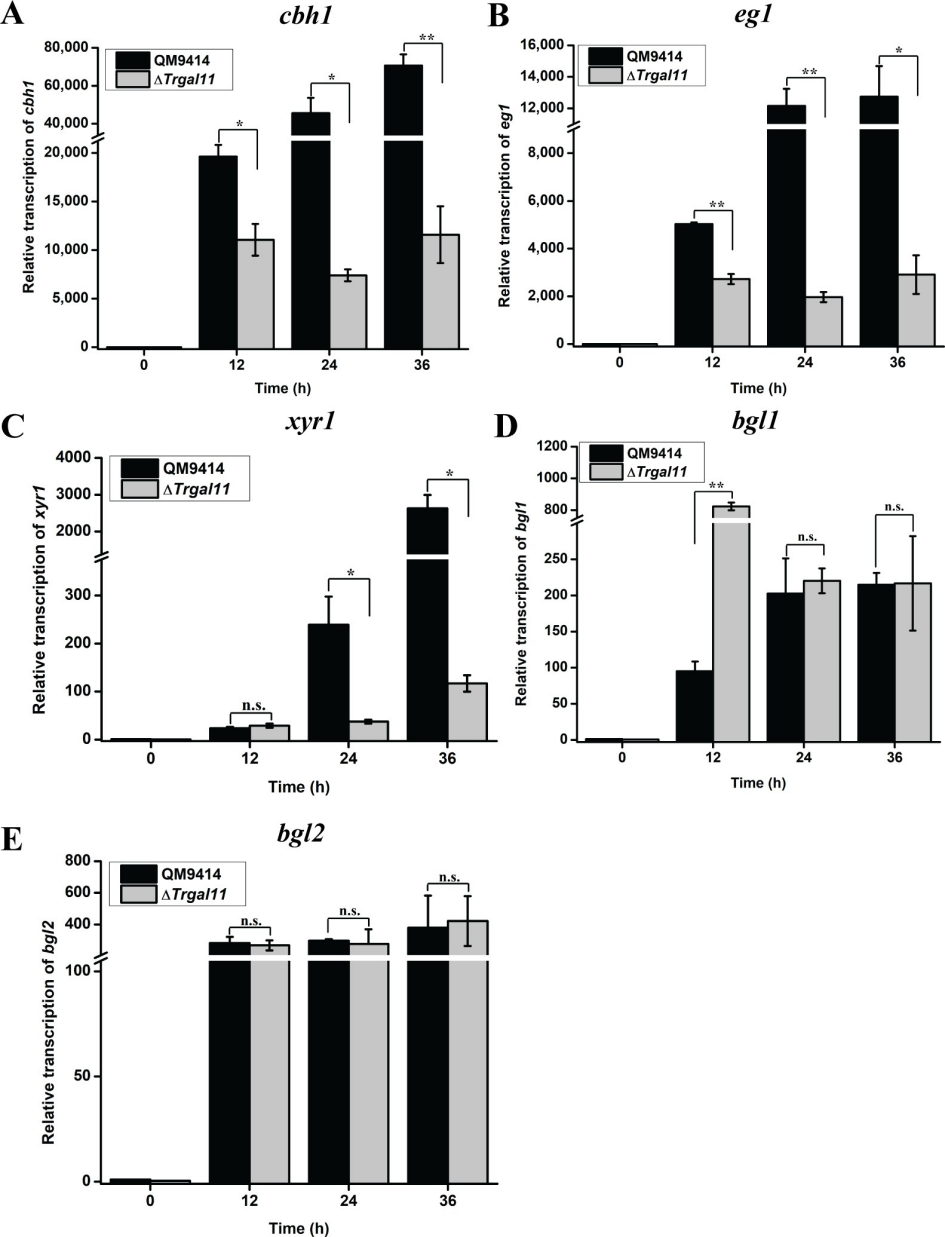
Deletion of *Trgal11* resulted in a significant decrease in the transcription of cellobiohydrolase and endoglucanase but not β-glucosidase genes. Transcription of *cbh1* (A), *eg1* (B), *xyr1* (C), *bgl1*(D), and *bgl2* (E) were analyzed by quantitative RT-PCR after induction on 1% (w/v) Avicel. The expression level of the *actin 1* gene was used as a reference gene for normalization in all samples. Significant differences (t-test, *P<0.05, **P<0.01) were detected for *cbh1*, *eg1*, and *xyr1* gene transcription between QM9414 and Δ*Trgal11* for the indicated time points after induction. No significant differences (t-test, P>0.05, n.s.) were detected for *bgl1* and *bgl2* gene transcription between QM9414 and Δ*Trgal11* for the indicated time points after induction except *bgl1* gene transcription induction for 12 h.

To determine the role of TrGAL11 in cellulase gene expression, extracellular cellulase and hemicellulase activities of the Avicel-induced cultures of three independent Δ*Trgal11* deletion strains were analyzed. *Trgall1* deletion resulted in an up to 50~60% reduction in extracellular cellobiohydrolase, CMCase, filter paper activities as well as a 55~70% reduction in total protein compared to the parent strain QM9414 (Fig 3A–3D and 3F). The absence of TrGAL11 also resulted in an up to 70% reduction in the induced expression of xylanases by xylan (S5 Fig). Unexpectedly, *p*NPG hydrolytic activities as determined for β-glucosidases were hardly affected (Fig 3E), indicating that TrGAL11 was differentially involved in the induced expression of most (hemi)cellulase versus β-glucosidase genes in *T*. *reesei*. Further examination of endogenous *cbh1*, *eg1*, *bgl1*, and *bgl2* mRNA levels by RT-qPCR demonstrated that the decreased cellulase activities as observed in the deletion strain were resulted from a down-regulation in the steady state transcripts of these cellulase as well as the *xyr*1 genes (Fig 4A–4C). In accordance with the hydrolytic activities, the transcription of two major β-glucosidase genes (*bgl1* and *bgl2*) were hardly affected (Fig 4D and 4E). Altogether, the data indicate that *Trgal11* plays an important role in mediating the induced expression of cellobiohydrolase and endoglucanase but not β-glucosidase genes in *T*. *reesei*.

To evaluate the relevant contribution of other putative tail module subunits to the induced cellulase gene expression, the identified *Trmed3*, *Trmed5*, and *Trmed16* that have been shown to be nonessential in yeast [43], were individually deleted in *T*. *reesei*. All these mutants displayed hardly any growth defect on minimum medium plates with four different carbon sources and only Δ*Trmed5* showed reduced conidiation compared to QM9414 (S6 Fig). Analysis of the extracellular hydrolytic activities revealed that, whereas *Trmed5* deletion, similar to *Trgal11*, resulted in a dramatic decrease in extracellular *p*NPC and filter paper but not *p*NPG hydrolytic activities (S7 Fig A-C), the absence of *Trmed3* or *Trmed16* hardly affected the induced biosynthesis of cellulases (S7 Fig D-J). Taken together, these data suggest that *T*. *reesei* Mediator may adopt a subtly different tail module organization from that reported for yeast to connect XYR1 to communicate the regulatory input to the Pol II enzyme.

### TrGAL11 is recruited to cellulase gene promoters in an XYR1-dependent manner

**Fig 5:**
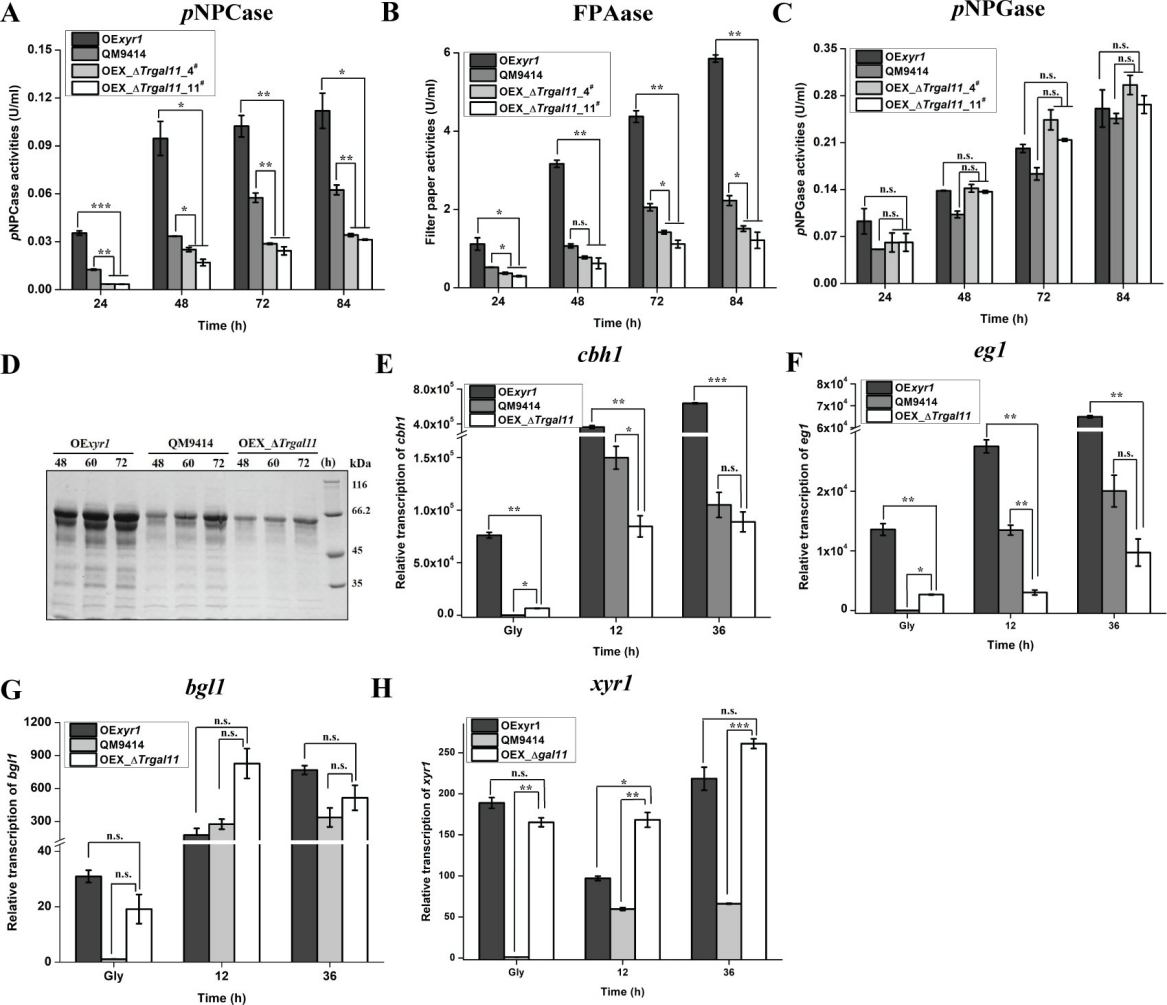
Overexpression of *xyr1* failed to restore the fully induced expression of cellulase genes in the *Trgal11* deletion mutant. (A-C): Extracellular *p*NPCase activity (A), FPAase activity (B), and *p*NPGase activity (C) of the culture supernatant from OE*xyr1*, QM9414 and two independent OEX_Δ*Trgal11* transformants on 1% (w/v) Avicel for the indicated time periods. Significant differences (*t*-test, *P<0.05, **P<0.01, ***P<0.001) were detected for the *p*NPCase activity, filter paper activities (FPAase) between OEX_Δ*Trgal11* and OE*xyr1* or between OEX_Δ*Trgal11* and QM9414. No significant difference (*t*-test, P>0.05; n.s.) was detected for the *p*NPGase activity. (D) SDS-PAGE analysis of the culture supernatant from the OE*xyr1*, QM9414 and OEX_Δ*Trgal11* strains cultured on 1% (w/v) Avicel. Coomassie Brilliant Blue was used for gel staining. (E-H): Transcription of *cbh1* (E), *eg1* (F), *bgl1* (G), and *xyr1* (H) were analyzed by quantitative RT-PCR after induction on 1% (w/v) Avicel. The expression level of the *actin* gene was used as a reference gene for normalization in all samples. Significant differences (*t*-test, *P<0.05, **P<0.01, ***P<0.001) were detected for the transcription of *cbh1*, *eg1*, and *xyr1* between OEX_Δ*Trgal11* and OE*xyr1* or between OEX_Δ*Trgal11* and QM9414. No significant difference (*t*-test, P>0.05; n.s.) was detected for *bgl1* transcription between OEX_Δ*Trgal11* and OE*xyr1* or between OEX_Δ*Trgal11* and QM9414.

To ask whether the significantly decreased expression of the *cbh*/*eg* genes in the Δ*Trgal11* strain was caused by the down-regulated *xyr1* transcripts, a recombinant strain that simultaneously overexpressed *xyr1* under control of the *tcu1* promoter in the Δ*Trgal11* strain (OEX_Δ*Trgal11*) was constructed. Extracellular hydrolytic activity and RT-qPCR analyses revealed that *xyr1* overexpression failed to restore the induced expression of cellulase genes (Fig 5). These results thus indicate that *xyr1* overexpression was insufficient to rescue the defective induction of cellulase gene expression without TrGAL11.

**Fig 6:**
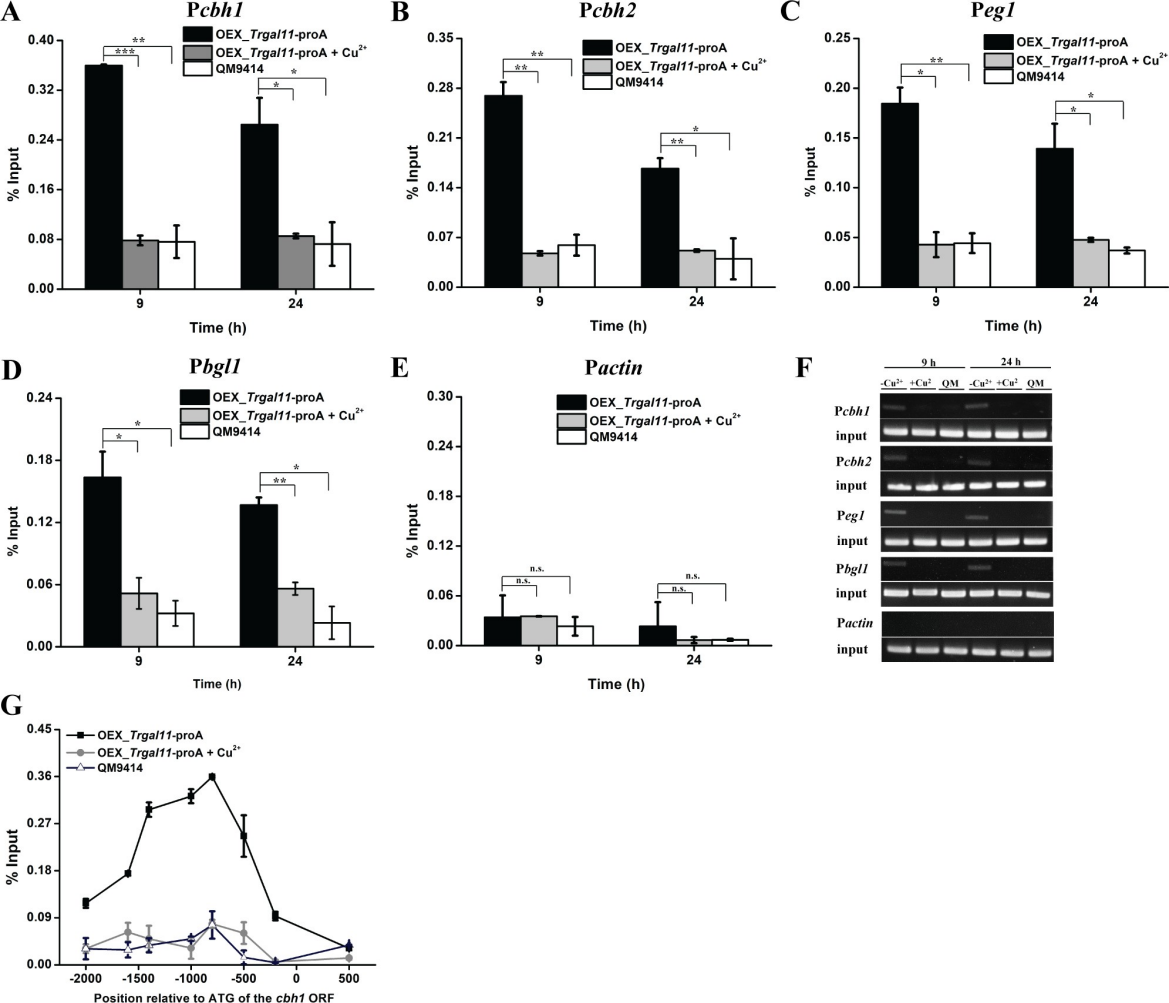
TrGAL11 was recruited to cellulase gene promoters in an XYR1-dependent manner. (A-E): ChIP assay for TrGAL11 binding to the *cbh1* (A), *cbh2* (B), *eg1* (C), *bgl1* (D), and *actin* (E) promoters in the OEX _Trgal11-proA strain after induction for 9 h and 24 h on 1% Avicel with or without adding exogenous copper. QM9414 was set as a negative control strain. IgG-agarose beads was used to immunoprecipitate TrGAL11-proA fusion protein bound to promoters. Significant differences (t-test, *P<0.05, **P<0.01, ***P<0.001) were detected for TrGAL11 binding to cellulase gene promoters when XYR1 was overexpressed without copper compared to with copper or QM9414 after Avicel induction. No significant difference (t-test, P>0.05, n.s.) was detected for TrGAL11 binding to the actin promoter regardless of the presence or absence of copper. (F) Semi-quantitative PCR products amplified from the above promoters with the immunoprecipitated DNA and resolved by agarose electrophoresis. -Cu^2+^, +Cu^2+^ and QM denoted samples from the OEX_Trgal11-proA strain cultured without and with copper as well as QM9414, respectively. (G) An overview of TrGAL11 occupancy over the *cbh1* promoter after Avicel induction for 9 h. The analyzed regions include *cbh1*-ORF (418 to 603), P*cbh1*-250 (-179 to -355), P*cbh1*-500 (-460 to -559), P*cbh1*-800 (-664 to -905), P*cbh1*-1000 (-952 to -1149), P*cbh1*-1400 (-1286 to -1427), P*cbh1*-1700 (-1603 to -1840), and P*cbh1*-2100 (-2112 to -2283). The numbers within brackets are the nucleotide position relative to the start codon ATG.

Considering the detected interaction between TrGAL11 and XYR1, we tested whether TrGAL11 is recruited by XYR1 to the cellulase gene promoter to directly participate in cellulase gene expression. To this end, we constructed a recombinant strain OEX_*Trgal11*-proA, wherein a C-terminal protein A-tagged endogenous TrGAL11 was expressed simultaneously with XYR1 under the control of the *tcu1* promoter. P*tcu1* is a highly sensitive copper repressive promoter which allowed the expression of *xyr1* either to be highly expressed without exogenous copper or to be shut off with copper [37]. Fusion of C terminal protein A tag with TrGAL11 hardly affected its normal function (S8 Fig). Chromatin immunoprecipitation (ChIP) followed by quantitative PCR (ChIP-qPCR) or semi-quantitative PCR was then performed to determine TrGAL11-proA occupancy on cellulase gene promoters. As shown in Fig 6A–6D and 6F, TrGAL11 was highly enriched on all the tested cellulase gene promoters including the *bgl1* promoter when *xyr1* was expressed without exogenous copper. TrGAL11 recruitment was, however, dramatically decreased if the strain was cultured in the presence of copper wherein the XYR1 expression was repressed. As expected, no significant enrichment of TrGAL11 was detected on the *actin* promoter regardless of the expression of XYR1 (Fig 6E and 6F). An overview of TrGAL11 occupancy over the whole *cbh1* promoter further revealed that TrGALl1 was significantly more enriched on a promoter region around -800 bp upstream of the start codon, where multiple XYR1 binding sites have been reported [44]. Again, TrGAL11 occupancy strictly depends on XYR1 as seen with the sharply decreased TrGAL11 occupancy signals all over the promoter region when *xyr1* was turned off with copper (Fig 6G).

**Fig 7:**
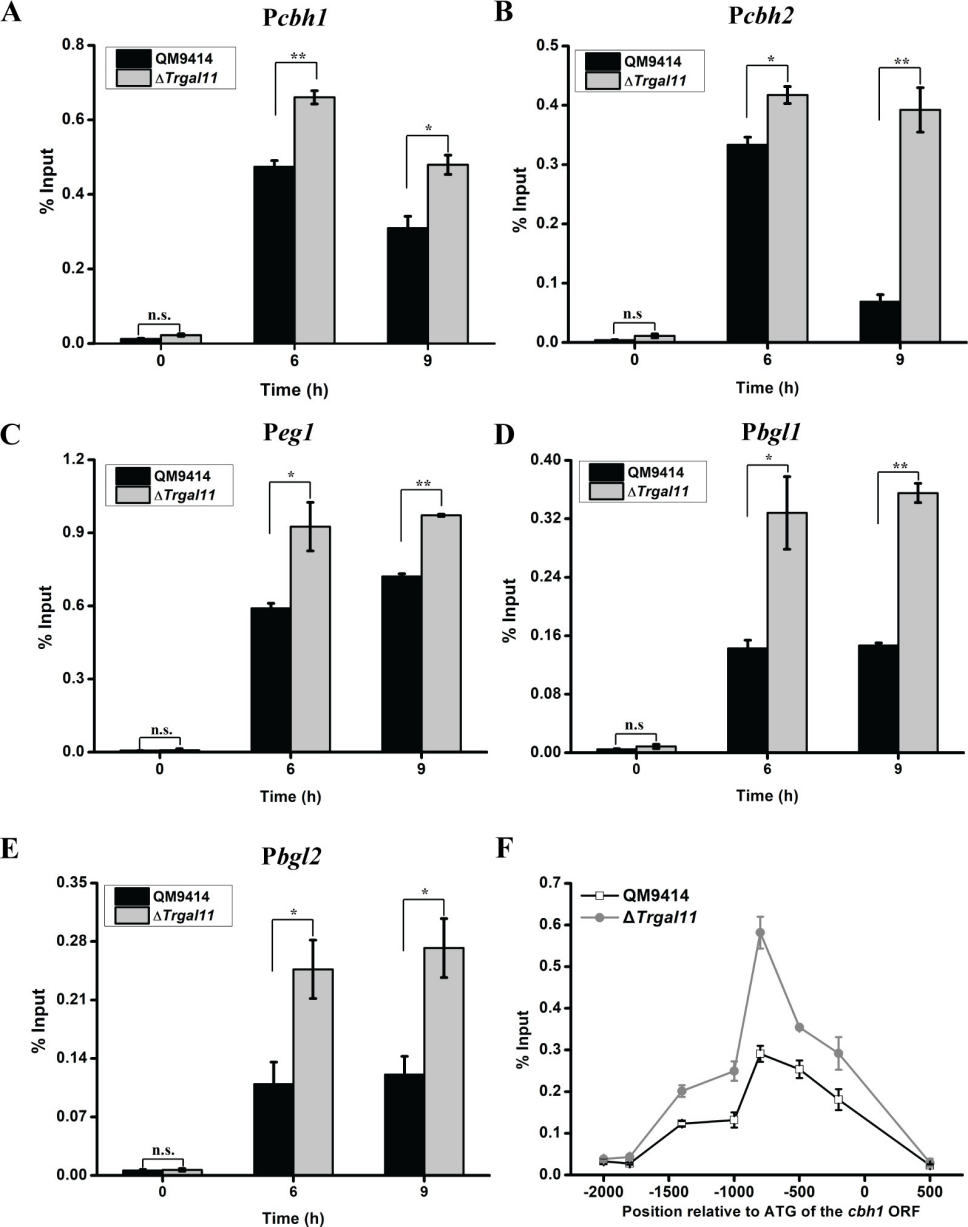
*Trgal11* deletion resulted in a significantly higher XYR1 occupancy on cellulase gene promoters. (A-E): ChIP assay of XYR1 binding to *cbh1*(A), *cbh2* (B), *eg1*(C), *bgl1*(D), and *bgl2* (E) promoters of QM9414 and the Δ*Trgal11* mutant after induction for 6 h and 9 h on 1% Avicel. Anti-XYR1 antibody was used to immunoprecipitate XYR1 bound to all detected cellulase gene promoters. Significant differences (*t*-test, *P<0.05, ** P<0.01) were detected for XYR1 occupancy on cellulase gene promoters between QM9414 and Δ*Trgal11* mutant after Avicel induction for 6 h and 9 h. (F) An overview of XYR1 occupancy over the *cbh1* promoter analyzed by ChIP after Avicel induction for 9 h. The analyzed regions are defined as in Fig 6G.

Given the facts that *xyr1* transcripts were significantly decreased with *Trgal11* deletion and that XYR1 overexpression failed to rescue the induction defect, we asked whether *Trgal11* deletion affected XYR1 binding to cellulase gene promoters. ChIP-qPCR analyses revealed significantly higher XYR1 occupancy on all the relevant cellulase gene promoters including β-glucosidase gene promoters in the *Trgal11* deletion strain than that in QM9414 (Fig 7A–7E). A further overview of XYR1 occupancy over the *cbh1* promoter showed a similar binding pattern to that of TrGALl1 (Fig 7F). The enrichment signals for XYR1 in the absence of TrGAL11 were significantly higher than those of QM9414 at regions from -250 to -1500 bp upstream of ATG. Taken together, these results indicate that TrGAL11 and potentially Mediator recruitment to cellulase gene promoters specifically depends on XYR1, most probably through its direct interaction with XYR1. The results also imply that the recruited Mediator or activated PIC may somehow regulate the dynamic binding of XYR1 to its target promoters.

### TrGAL11 contributes to RNA Pol II recruitment to cellulase gene core promoters

**Fig 8:**
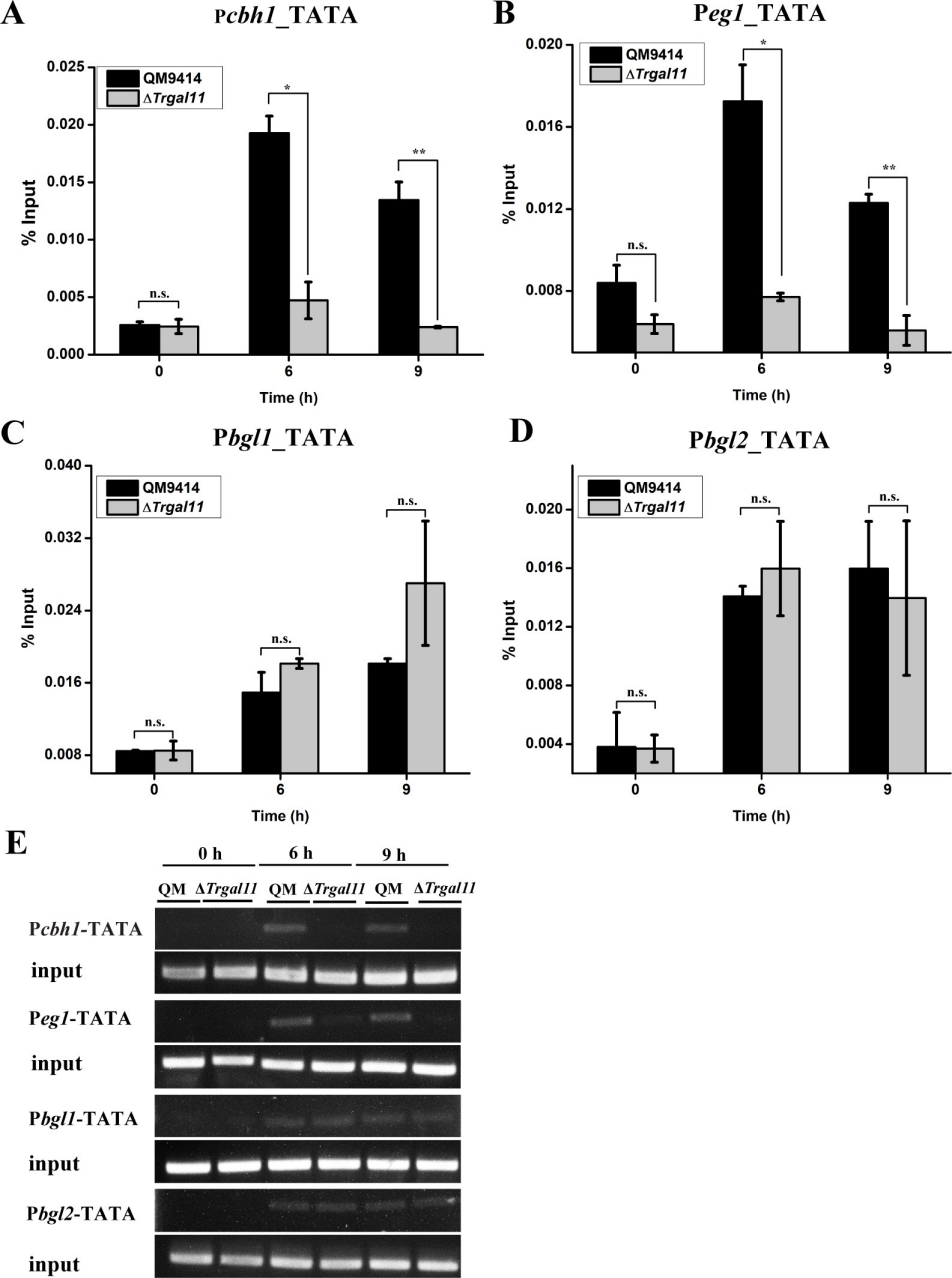
TrGAL11 plays a critical role in RNA Pol II recruitment on the core-promoter of *cbh*/*eg* genes but not of *bgl* genes. ChIP assay of RNA Pol II occupancy on the core promoters (TATA box) of the *cbh1* (A), *eg1* (B), *bgl1* (C), and *bgl2* (D) genes of QM9414 and Δ*Trgal11* under 1% Avicel induction for 6 h and 9 h. Anti-Rpb1 CTD antibody was used to immunoprecipitate Rpb1 bound to cellulase gene core promoters. Significant differences (*t*-test, *P<0.05, **P<0.01) were detected for RNA Pol II occupancy on *cbh1* and *eg1* core promoters after induction for 6 h and 9 h but no significant differences (*t*-test, P>0.05, n.s.) were detected on *bgl1* and *bgl2* core promoters. (E) Semi-quantitative PCR products amplified from the above core promoter regions from the immunoprecipitated DNA and resolved by agarose electrophoresis.

As the Mediator tail module mainly functions to connect sequence-specific transcription factors to promote the formation of PIC, it was reasonable to believe that the absence of TrGAL11 would compromise RNA Pol II recruitment to the core promoter upon cellulase gene activation. To test this hypothesis, ChIP-qPCR was performed in the Δ*Trgal11* and QM9414 strains with an antibody against RNA Pol II subunit Rpb1 C-terminus domain (CTD) (8WG16) to analyze RNA Pol II occupancy on cellulase gene core promoter containing the TATA box. As shown in Fig 8A and 8B, significant Rpb1 recruitment was detected on *cbh*/*eg* cellulase gene core promoters upon Avicel induction in QM9414, which was in contrast with the dramatically decreased Rpb1 binding in the Δ*Trgal11* strain. In accordance with transcription analyses, no significant difference in Rpb1 recruitment was observed for *bgl1* and *bgl2* core promoters (Fig 8C and 8D). The differential recruitment of RNA Pol II to *cbh*/*eg* and *bgl* core promoters in the presence or absence of TrGAL11 was further demonstrated by semi-quantitative PCR using the same immunoprecipitated DNA as that for ChIP-qPCR (Fig 8E). Together, these data suggest that TrGAL11 plays an important role in recruiting RNA Pol II to cellobiohydrolase and endoglucanase gene core promoters upon cellulose induction, most probably followed by its direct interaction with XYR1.

## Discussion

In this study, we showed that the major transactivator XYR1 directly targets the Mediator tail module subunit TrGAL11 to initiate (hemi)cellulase gene transcription in *T*. *reesei*. Tail module subunits including TrGAL11 and TrMED5 were demonstrated to be differentially involved in the cellulase gene expression. In addition, TrGAL11 and thus RNA Pol II binding to cellulase gene promoters specifically relied on the expression of XYR1 whereas the steady state XYR1 binding to its target regulatory sequences was subject to a regulation imposed by TrGAL11 recruitment.

Regulation of eukaryotic Pol II transcription is carried out in many ways, from the DNA sequence and chromatin architecture to recruitment and regulation of large protein assemblies at the promoter [45]. Central to this regulation is the multisubunit Mediator complex, which has been established as an essential involvement in communicating regulatory inputs from specific DNA-binding transcription factors and promoter-bound complexes directly to the Pol II enzyme [46]. In doing so, Mediator functions to facilitate the formation of the so-called preinitiation complex (PIC), which assembles at transcription start sites and regulates Pol II recruitment and activity [47]. Unlike core mediator composed of head and middle modules, the tail domain of Mediator remains unresolved at higher resolution due to its conformational heterogeneity [7, 48]. While it is generally believed that MED2, MED3, and MED15/GAL11 subunits can form a stable triad separable from the rest of Mediator, MED15/GAL11 also interacts with MED16 to stabilize its interaction, as well as that of MED5, with the tail [48]. Our observation that TrGAL11 but not TrMED3 deletion impaired the induced *cbh*/*eg* cellulase gene expression is consistent with previous genetic analyses of the functions of the triad that revealed distinct *in vivo* phenotypes associated with individual deletions of these tail module subunits in yeast [19]. The fact that the MED2 ortholog encoding gene was not readily identified in *T*. *reesei* may lie in the possibility that an ortholog distantly related to this yeast counterpart does exist which awaits further identification. Built upon these data, we found that TrMED16 deletion hardly affected the induced cellulase gene expression although the entire Tail module would be destabilized and lost if the y*med16* gene was deleted [48]. This role of yMED16 has been attributed to its contact with the scaffold subunit yMED14. Nonetheless, the phenotypic difference may be reconciled by the facts that yeast cells that lack yMED16 show no defects in the induced expression levels of two GCN4-dependent genes even though the middle or head module Mediator subunits cannot be detected at these promoters [12]. Moreover, evidence exists that yMED16 depletion does not lead to a parallel loss of yMED15 [24]. On the other hand, the observed effect of TrMED5 on cellulase gene expression is in accordance with the note that the N-domain of MED5 can interact with MED15/GAL11 to form a tetrameric complex with the MED2-MED3-MED15/GAL11 triad in the absence of MED16 [49]. Altogether, these results may otherwise implicate that *T*. *reesei* Mediator may adopt a tail organization subtly different from those reported when specifically acting at cellulase genes.

Among the various transcriptional regulators involved in controlling cellulase gene expression, XYR1 is so far the most important transcriptional activator, which is absolutely required for expression of both xylanase and cellulase-encoding genes [30]. In the present study, we demonstrated that XYR1 AD interacts with the KIX domain of TrGAL11 *in vitro* and TrGAL11 recruitment to cellulase gene promoters specifically depends on XYR1. The following recruitment of RNA PolⅡ at cellobiohydrolase and endoglucanase gene core promoters was then shown to be severely compromised in the absence of TrGAL11. Of particular note, the transcription of most (hemi)cellulase genes and two major β-glucosidase genes displayed a differential requirement for the Tail module subunits including TrGAL11 and TrMED5. While ample evidence exists that the mediator tail domain, especially the MED15/GAL11 subunit, serves as an important target of various transcriptional activators [11, 12, 20, 39, 40], it is reasonable to believe that multiple interactions involving different tail subunits would ensure the efficient Mediator recruitment. Loss of interactions with specific tail module subunits may thus have different effects in the regulation of a subset of genes. In this respect, it has been reported that, while disrupting the interaction between yGAL11 and yGAL4 impairs transcription activation, deletions of yGAL11, yMED3, or yMED2 had little effect on transcriptional activation by yGCN4 *in vivo* although both activators has been found to interact with yGAL11 [15, 16]. One possible explanation for the differential involvement of TrGAL11 and TrMED5 in the cellulase gene expression is that an as yet to be identified transcriptional activator synergizes with XYR1 in achieving core Mediator recruitment at β-glucosidase gene promoters by engaging interactions with subunits other than TrGAL11 and TrMED5.

An interesting finding in our present research was that XYR1 binding to cellulase gene promoters seems to be significantly enhanced although *xyr1* expression itself was reduced in the absence of TrGAL11. The quantitatively bound XYR1, however, is incapable of activating the full expression of cellulase genes except that of β-glucosidase genes without TrGAL11. On the one hand, these results reinforce the point that TrGAL11 and potentially the so-called core Mediator including the head and middle modules play a critical role in mediating the XYR1-activating cellulase gene transcription. On the other hand, the data implicate that the steady state XYR1 occupancy on cellulase gene promoters seem to be subject to a feedback regulation exerted by recruited Mediator or activated PIC. In analogy with recent studies showing that a growing number of transcriptional regulators are subject to the control of ubiquitin-proteasome system (UPS) either by an “activation by destruction” mechanism to destroy them when their function is no longer appropriate or by processing them into a functional state via limited proteolysis [50, 51]. One can assume that XYR1 may also undergo a similar process restricting its function when it is not appropriate during cellulose induction. Notwithstanding with this, the precise mechanism involved in this potential regulation warrants further study.

**Fig 9:**
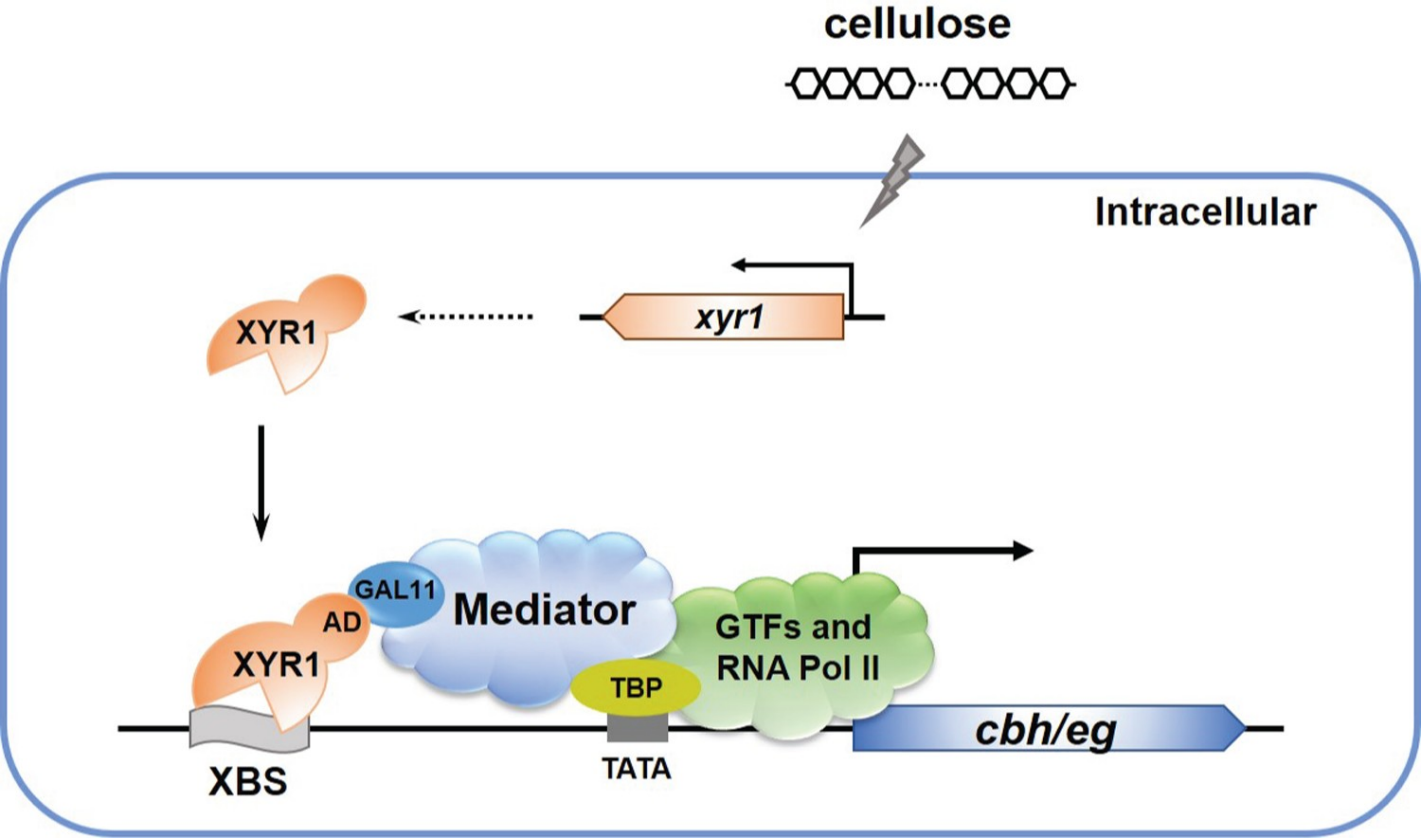
A schematic model of how Mediator is recruited by XYR1 to participate in activating cellulase gene expression in *T*. *reesei*. Once expressed upon induction, XYR1 binds to the upstream binding sites (XBS) in cellulase gene promoters and recruits the Mediator complex by interacting with the tail module subunit TrGAL11 to further facilitate the recruitment of the general transcription machinery including RNA Pol II to *cbh* and *eg* genes.

In summary, a working model based on the present data is provided in Fig 9. Upon cellulose induction, the key transcriptional activator XYR1 binds to its binding sites within cellulase gene (*cbh*/*eg*) promoters and recruits the Mediator complex through a direct interaction with the tail subunit TrGAL11, which further facilitates the recruitment of the general transcription machinery including RNA Pol II to successfully initiate the transcription of these cellulase genes. In contrast with *cbh* and *eg* genes, there likely exists an as yet to be identified transcriptional activator, which synergizes with XYR1 to achieve core Mediator recruitment to β-glucosidase gene promoters by engaging interactions with subunit(s) other than TrGAL11.

## Materials and methods

### Strains and cultivation condition

*Escherichia coli* DH5α cells were used for plasmids construction and *E*. *coli* Origami BL21 (DE3) cells were used as a host for the production of the recombinant proteins. Both strains were cultured in lysogeny broth with a rotary shaker (200 rpm) at 37°C.

The *S*. *cerevisiae* strain Y2H Gold (*MATa*, *ura3-52*, *his3-200*, *ade2-101*, *trp1-901*, *leu2-3*, *112*, *gal4*Δ, *gal80*Δ, LYS2::*GAL1*_*UAS*_-*Gal1*_*TATA*_-His3, *GAL2*_*UAS*_-*Gal2*_*TATA*_*-Ade2URA3*::*MEL1*_*UAS*_-*Mel1*_*TATA*_ *AUR1-C MEL1*) was used as the host for the two-hybrid screen. Yeast cells were routinely cultivated at 30°C in YPD medium (1% yeast extract, 2% peptone, and 2% glucose). Synthetic complete (SC) medium lacking tryptophan, leucine, histidine, and adenine with 75 ng/mL of AbA was used for transformant selection. For plate growth assays, serial dilutions of yeast cell suspensions were spotted onto selective plates containing 75 ng/mL of AbA that were allowed to grow at 30°C.

*T*. *reesei* QM9414 (ATCC 26921) and QM9414Δ*pyr4* in which the uridine trophic marker gene was deleted in QM9414 [52] were used throughout this work as control and parental strains, respectively. All *T*. *reesei* strains were maintained on malt extract agar supplemented with 10 mM uridine when necessary. For the transcription and (hemi)cellulase production analyses, *T*. *reesei* strains were pre-grown in 1 L Erlenmeyer flasks on a rotary shaker (200 rpm) at 30°C in 250 mL Mandels-Andreotti (MA) medium with 1% (v/v) glycerol as the carbon source for 48 h as previously described [53]. Mycelia were harvested by filtration and washed twice with medium without a carbon source. Equal wet weight (4 g) of mycelia were then transferred to fresh medium without peptone containing 1% (w/v) Avicel or other carbon sources as indicated, and incubation was continued for the indicated time periods.

### Plasmids and recombinant strains construction

To verify the interaction of XYR1 and TrGAL11 in yeast two hybrid, the full length and KIX domain (amino acids 1~134) of TrGAL11 were amplified from *T*. *reesei* cDNA and were inserted into the pGADT7 vector after digestion with *Nde*I and *Bam*HI to obtain the pGADT7-TrGAL11 FL and pGADT7-TrGAL11 KIX plasmids. The pGBKT7-XYR1 AD was constructed as described previously [54]. To delete *Trgal11*, two DNA fragments corresponding to approximately 2.2 kb of *Trgal11* up- and downstream non-coding regions were amplified from QM9414 genomic DNA and ligated into pDONOR*pyr4* [53] via BP-cloning (Invitrogen) to yield the disruption vector pDONOR*Trgal11pyr4*, which was used to transform *T*. *reesei* QM9414Δ*pyr4* and OE*xyr1* [33] strains after linearization with I-*Sce*I to obtain the Δ*Trgal11* and OEX_Δ*Trgal11* strains, respectively. Similarly, to delete *Trmed5*, *Trmed3* or *Trmed16*, approximately 2.2 kb of *Trmed3* up- and 2.0 kb of downstream non-coding regions or 2.0 kb of *Trmed5* and *Trmed16* up- and downstream non-coding regions were amplified from QM9414 genomic DNA and ligated into pDONOR*pyr4* via BP-cloning to yield the pDONOR*Trmed5pyr4*, pDONOR*Trmed3pyr4* and pDONOR*Trmed16pyr4* disruption vectors, respectively, which was used to transform *T*. *reesei* QM9414Δ*pyr4* after linearization with I-*Sce*I to obtain the Δ*Trmed5*, Δ*Trmed3*, and Δ*Trmed16* strains, respectively.

For construction of the strain expressing a protein A-tagged TrGAL11, the *trp*C terminator was amplified from pMDP*tcu1*-T*trpC* [37], digested with *Hin*dIII/*Asc*I, and then ligated into pUC19-*pyr4* [55] to obtain pUC19-*pyr4*-T*trpC*. The protein A tag encoding sequence was amplified from the pMDP*tcu1*proA-*Trswi1* plasmid [56], digested with *Not*I/*Pme*I, and subsequently ligated into the pUC19-*pyr4*-T*trpC* plasmid to obtain the knock-in plasmid pUC19-*pyr4*-proA. Finally, a 2 kb fragment upstream from the stop codon of the *Trgal11* gene and a 2.3 kb fragment downstream from TGA were amplified from QM9414 genomic DNA, digested with *Hin*dIII/*Not*I and *Xba*I/*Spe*I respectively, and then ligated into pUC19-*pyr4*-proA to fuse the *Trgal11* with protein A coding sequence to obtain the pUC19-*Trgal11*-proA-KI plasmid. The plasmid was linearized with *Spe*I prior to being transformed into the OE*xyr1* strain to obtain OEX_*Trgal11*-proA.

*T*. *reesei* transformation was carried out essentially as previously described [55]. The transformants were selected on minimal medium for either uridine prototroph or for resistance to hygromycin (120 μg/mL). Anchored PCR was used to verify the correct integration events. All *T*. *reesei* strains used in this research were listed in S3 Table.

### Vegetative growth and conidiation analyses

To assay vegetative growth, strains were precultured on minimal media agar plate for two days and then a slice of agar with the same area of growing mycelia of the corresponding strain (1 cm in diameter) was taken from the plate and inoculated on minimal media agar plates containing different carbon sources (glucose, glycerol, cellobiose or lactose) at 30°C for 3 days or on malt extract agar plates incubated for 5 days.

To determine *T*. *reesei* biomass accumulation in liquid MA medium with 1% (w/v) glucose or Avicel as the sole carbon source, equal amounts of pre-cultured mycelia as determined by wet weight (4 g) were inoculated into the indicated medium. The mycelia collected at growth intervals were either dried at 70°C for 48 h and then weighed for mycelia grown on glucose or broken for determining intracellular protein content [57].

### Enzyme activity and protein analysis

Cellulolytic enzyme activity was determined as previously described [55, 58]. Briefly, cellobiohydrolase and β-glucosidase activities were determined by measuring the amount of released p-nitrophenol using *p*-nitrophenyl-D-cellobioside (*p*NPC; Sigma) and *p*-nitrophenyl-β-D-glucopyranoside (*p*NPG; Sigma) as the substrates, respectively. The cellulase activity assays were performed in 200 μL reaction mixtures containing 50 μL of culture supernatant and 50 μL of the respective substrate plus 100 μL of 50 mM sodium acetate buffer (pH 4.8) and then incubated at 45°C for 30 min [55]. One unit (U) of *p*NPCase activity is defined as the amount of enzyme releasing 1 μmol of pNP per minute. The endo-glucanases and filter paper activities (FPAase) were determined by measuring the released reducing sugar with carboxymethylcellulose sodium salt (CMC; Sigma) and filter paper as substrates, respectively. Determination of CMC hydrolytic activities was carried out at 50°C in a 100 μL reaction mixture containing 50 μL of appropriately diluted culture supernatant and 50 μL of 0.5% (w/v) CMC sodium in 50 mM sodium acetate buffer (pH 4.8). The FPA assay was performed at 50°C in a 200 μL reaction mixture including 50 μL of appropriately diluted culture supernatant and 150 μL 50 mM sodium acetate buffer (pH 4.8) with Whatman No. 1 filter paper as substrates. One unit (U) of CMCase or FPA was defined as the release of 1 μmol reducing sugar per minute under the test conditions. Xylanase activities were determined by measuring the amount of released xylose using xylan as substrate. Briefly, a reaction mixture containing 60 μL of diluted culture supernatant and 60 μL of beechwood xylan (5 g/L) dissolved in 50 mM sodium acetate buffer (pH 4.8) was incubated at 50°C for 15 min. The reducing sugar released in the mixture was determined using DNS method with xylose as the standard. One unit of enzyme activity was defined as the amount of enzyme capable of releasing 1 μmol of xylose per minute [53]. Total secreted and intracellular proteins were determined using the Bradford protein assay with bovine serum albumin (BSA) as a standard.

### Quantitative RT-PCR (qRT-PCR)

Total RNAs were extracted using the TRIzol reagent (Invitrogen, Grand Island, NY, USA) and purified using the TURBO DNA-free kit (Ambion, Austin, TX, USA) to eliminate genomic DNA contamination according to the manufacturer’s instructions. Reverse transcription was performed using the PrimeScript RT reagent Kit (Takara, Japan) according to the instructions. Quantitative PCR was performed using SYBR green supermix (TaKaRa) on a Bio-Rad myIQ5 thermocycler (Bio-Rad). Data analysis was performed using the relative quantitation/comparative CT (ΔΔCT) method and were normalized to an endogenous control (*actin*), with expression on glycerol as the reference sample. Three biological replicates were performed for each analysis and the results and errors are the mean and SD, respectively, from the replicates. Statistical analysis was performed using the student’s *t*-test analysis.

### Chromatin immunoprecipitation (ChIP) analyses

ChIP assays were performed according to a previously described protocol [33, 59]. Briefly, the mycelia were fixed in minimal medium containing 1% formaldehyde at 30°C for 10 min with shaking before the cross-linking was quenched via the addition of 25 mL of 1.25 M glycine for 5 min. The mycelia were then collected, ground in liquid N_2_ and broken in lysis buffer (50 mM HEPES pH 7.5, 150 mM NaCl, 1 mM EDTA, 0.5% Triton X-100, 0.1% sodium deoxycholate, 0.1% SDS, 1 mM PMSF (phenylmethanesulfonyl fluoride), 1 μg/mL leupeptin, and 1 μg/mL pepstatin) with glass beads (0.45 mm). This crude lysate was further sonicated to obtain an average DNA fragment size of approximately 500 bp. Immunoprecipitation was performed by incubating IgG (GE Healthcare), anti-Rpb1 CTD (8WG16, abcam) or anti-XYR1 antibody [33], with an aliquot of the clarified cell lysates containing equal amounts of protein (2 mg) at 4°C for 5 h. Forty microliters of protein A/G beads pre-coated with 1 mg/mL of BSA and 1 mg/mL of fish sperm DNA were used per IP. Following immunoprecipitation and extensive sequential washes, the DNA was eluted with elution buffer (100 mM Tris-HCl (pH 7.8), 10 mM EDTA, 1% SDS, 10 mM NaHCO_3_, and 100 mM NaCl) at 65°C for 5 h and recovered by proteinase K treatment of the pelleted samples at 45°C for 1 h, phenol-chloroform extraction and ethanol precipitation. Quantitative PCR was performed with the input and the precipitated chromatin DNAs using a Bio-Rad IQ5 thermocycler (Bio-Rad) and the SYBR Green Supermix (Takara). Relative enrichment of the DNAs was calculated as a percentage of the input DNA according to the RT-qPCR analysis. Twenty-eight or thirty-two cycles of semi-quantitative PCR amplification was also performed with the input and the precipitated chromatin DNA sample, respectively, followed by agarose gel electrophoresis [60]. All primers used for amplification in ChIP assays were listed in S2 Table and the corresponding promoter regions amplified by these primers were shown in S9 Fig. An excel file containing the original numerical data for ChIP-qPCR were included as S1 Data.

### Protein production in *E*. *coli* and GST pull down assays

For the expression of TrGAL11 KIX (amino acids 1~134) and XYR1 AD in *E*. *coli*, the DNA fragment coding for TrGAL11 KIX was amplified from the *T*. *reesei* cDNA and was inserted into the pET28a (+) expression vector after digestion with *Nde*I and *Not*I to obtain the pET28a-TrGAL11 KIX plasmid. Similarly, the XYR1 AD (amino acids 767~860) was amplified from the pGBKT7-XYR1 AD plasmid [56] and ligated into the pGEX4T-1 expression vector after digestion with *Not*I and *Bam*HI. To purify the GST-XYR1 AD_767-860_ and TrGAL11 KIX-His, the indicated expression constructs were transformed into CaCl_2_-treated competent *E*. *coli* BL21 (DE3) cells. Protein purification was carried out essentially as previously described [33]. All of the protein preparations were stored at -80°C in the presence of 20% (v/v) glycerol. GST pull-down assay was carried out as previously described [54]. Briefly, purified GST or GST-XYR1 AD_767-860_ pre-coupled on glutathione beads was incubated with TrGAL11 KIX-His and rotated for at least 2 h at room temperature. The supernatant was removed and the beads were washed three times with PBST (137 mmol/L NaCl, 2.7 mmol/L KCl, 10 mmol/L Na_2_HPO_4_, 2 mmol/L KH_2_PO_4_, 0.5% Triton X-100, pH 7.4). The proteins retained on the beads were resolved by SDS-PAGE and detected by Western blot with anti-His antibody (Sigma).

### Sequence analysis

Amino acid sequences from *T*. *reesei* and other relevant species were obtained from the NCBI (https://www.ncbi.nlm.nih.gov/) or JGI (https://genome.jgi.doe.gov/) databases.

### Statistical analysis

Statistical analysis was performed using the Student’s t test analysis. At least two or three biological replicates were performed for each analysis and the results and errors are the mean and SD, respectively, of these replicates.

## Supporting information

S1 Table *T*. *reesei* orthologs of the *S*. *cerevisiae* Mediator complex subunits. *T*. *reesei* homologs were listed based on their evolutionary similarity to the corresponding *S*. *cerevisiae* Mediator complex subunits. N/A: Not Available.
(DOCX)

S2 Table ChIP-qPCR primers used in this research. (DOCX)

S3 Table Strains used in this research. (DOCX)

S1 Fig Protein sequence alignment of TrGAL11 and ScGAL11. (A) Protein sequence alignment was performed by the Multiple Sequence Alignment tool MUSCLE (https://www.ebi.ac.uk/Tools/msa/muscle/) with the primary amino acid sequence of TrGAL11 and ScGAL11. (B) Secondary structural simulation of TrGAL11 KIX domain via SWISS-MODEL (https://swissmodel.expasy.org/) and structural comparison with ScGal11 KIX domain (PDB_2k0n).
(TIF)

S2 Fig Cartoon of the *S*. *cerevisiae* Mediator complex. Compositional organization of the *S*. *cerevisiae* Mediator complex was modified from references [5, 61].
(TIF)

S3 Fig Verification of the construction of the Δ*Trgal11* strain via diagnostic PCR. (A) Schematic illustration of the homologous integration of the *T*. *reesei pyr4* gene at the *Trgal11* locus resulting in the deletion of the coding sequences of *Trgal11*. (B) Diagnostic PCR was performed to verify the correct integration of the *pyr4* gene at the *Trgal11* locus. Lanes 1–3, the genomic DNA from three independent transformants was used as template; NC, the QM9414 genomic DNA was used as template.
(TIF)

S4 Fig *Trgal11* deletion resulted in an elevated resistance to hygromycin B and pyrithiamine. Growth of QM9414 and Δ*Trgal11* strains on MM plates with hygromycin B or pyrithiamine. The result shown represented one of at least two independent experiments.
(TIF)

S5 Fig *Trgal11* deletion reduced xylanase expression induced by xylan. Xylanase activity of the supernatant from the parental strain QM9414 and Δ*Trgal11* cultures on 1% (w/v) xylan for the indicated time periods. Significant differences (*t*-test, *P<0.05, **P<0.01, ***P<0.001) were detected for the extracellular activities between QM9414 and Δ*Trgal11* for the indicated time points after induction.
(TIF)

S6 Fig Deletion of *Trmed5*, *Trmed3*, and *Trmed16* hardly affect vegetative growth on minimal medium with different carbon sources. Growth and conidiation analysis of QM9414 and *Trmed5*, *Trmed3*, or *Trmed16* deletion strains on plates with various carbon sources at a final concentration of 1% (w/v) at 30°C for 3 days or on malt extract agar for 5 days. Conidiation was neither significantly compromised except *Trmed5* disruption.
(TIF)

S7 Fig Effect of Δ*Trmed5*, Δ*Trmed3*, and Δ*Trmed16* deletions on cellulase gene expression induced on Avicel. Extracellular *p*NPC (A, D, H), *p*NPG (B, E, I), and filter paper activities (FPA) (C, F, J) of the supernatant from the parental strain QM9414 and three independent transformants of Δ*Trmed5*, Δ*Trmed3*, or Δ*Trmed16* cultured on 1% (w/v) Avicel were determined for the indicated time periods, respectively.
(TIF)

S8 Fig Fusion of the protein A tag with TrGAL11 in the OE*xyr1* strain hardly affected its normal function. Extracellular *p*NPC activity of the supernatant from OE*xyr1* and two independent OEX_*Trgal11*-proA transformants cultured on 1% (w/v) Avicel for the indicated time periods. No significant differences (*t*-test, P>0.05, n.s.) were detected for the *p*NPC activities between OE*xyr1* and two independent OEX_*Trgal11*-proA transformants under induction condition.
(TIF)

S9 Fig Schematic demonstration of various cellulase gene promoters amplified with primers in ChIP-qPCR. The number below each short bar denotes the approximate position of the amplified promoter regions relative to the start codon ATG. XYR1 binding elements GGC(T/A)3 and the TATA box were labeled as vertical bars [44].
(TIF)

S1 Data ChIP-qPCR original numerical data for Figs 6–8. (XLSX)
