# Supplementary material for: Trichoderma reesei XYR1 activates cellulase gene expression via interaction with the Mediator subunit TrGAL11 to recruit RNA polymerase II
Source: PLoS Genet. 2020 Sep 2;16(9):e1008979. doi: 10.1371/journal.pgen.1008979 (PMC7467262; doi:10.1371/journal.pgen.1008979)
Supplement: S1 Table — T. reesei homologs were listed based on their evolutionary similarity to the corresponding S. cerevisiae Mediator complex subunits. N/A: Not Available. (DOCX) [file pgen.1008979.s001.docx]

**S1 Table. *T. reesei* orthologs of the *S. cerevisiae* Mediator complex subunits**

| Module | Yeast subunits  Universal name (Original name) | *T. reesei* orthologs | Identity |
| --- | --- | --- | --- |
| Head | MED6 | Tr4111 | 30.37% |
|  | MED8 | Tr78322 | 26.47% |
|  | MED11 | Tr75324 | 48% |
|  | MED17 (SRB4) | Tr103209 | 23.46% |
|  | MED18 (SRB5) | Tr111785 | 29.41% |
|  | MED20 (SRB2) | Tr66935 | 20.91% |
|  | Med22 (SRB6) | Tr121732 | 18.80% |
|  | MED19 (ROX3) | Tr53596 | 21.00% |
| Middle | MED9 (CSE2) | Tr42953 | 19.69% |
|  | MED10 (NUT2) | Tr62026 | 28.57% |
|  | MED21 (SRB7) | Tr55548 | 38.17% |
|  | MED31 (SOH1) | Tr65895 | 44.44% |
|  | MED7 | Tr82125 | 37.95% |
|  | MED4 | Tr2558 | 24.49 |
|  | MED1 | Tr122230 | 43.24% |
| Tail | MED14 (RGR1/HRS1) | Tr120618 | 22.86% |
|  | MED3 (PGD1) | Tr123911 | 21.72% |
|  | MED5 (NUT1) | Tr81882 | 20.73% |
|  | MED15 (GAL11) | Tr107300 | 23.88% |
|  | MED16 (SIN4) | Tr2666 | 20.47% |
|  | MED2 | N/A | N/A |
| Cyclin/Cdk | MED12 (SRB8) | Tr106037 | 28.31% |
|  | MED13 (SRB9/SSN2) | Tr22783 | 23.11% |
|  | CDK8 (SRB10) | Tr81720 | 56.63% |
|  | CycC (SRB11/SSN8) | Tr5196 | 32.26% |

*T. reesei* homologs were listed based on their evolutionary similarity to the corresponding *S. cerevisiae* Mediator complex subunits. N/A: Not Available.
