# Supplementary material for: Trichoderma reesei XYR1 activates cellulase gene expression via interaction with the Mediator subunit TrGAL11 to recruit RNA polymerase II
Source: PLoS Genet. 2020 Sep 2;16(9):e1008979. doi: 10.1371/journal.pgen.1008979 (PMC7467262; doi:10.1371/journal.pgen.1008979)
Supplement: S2 Table — (DOCX) [file pgen.1008979.s002.docx]

**S2 Table. ChIP-qPCR Primers used in this research**

| **Gene** | **Primer name** | **Sequence (5’🡪 3’)** |
| --- | --- | --- |
| *cbh1* | P*cbh1*-2100 qF  P*cbh1*-2100 qR  P*cbh1*-1700 qF  P*cbh1*-1700 qR  P*cbh1*-1400 qF | TCTACTTGTGGTCGCCTGCTA  TGACTCACTGATTGGGAACTCT  GTGCCATTCTTTTCCCTTCC  ATCCAGTTCTTCACCGTAGCC  CGAACCCGGAGAATCGAGATGTGC |
|  | P*cbh1*-1400 qR | GCTACTGCGACGGAACGCTTTGCT |
|  | P*cbh1*-1000 qF | CCTCTTCTCAACCTTTGGCGTTTC |
|  | P*cbh1*-1000 qR | TCTACGAGCAGAGTTCGGATAACA |
|  | P*cbh1*-800 qF | GGCAAGGGAAACCACCGAT |
|  | P*cbh1*-800 qR | TGGACTGAGTGAAGAAACAAACG |
|  | P*cbh1*-500 qF | GGCAGTGATGGAAGACAGTGAAA |
|  | P*cbh1*-500 qR | TCGTCGTATCGGCAGACAAACCT |
|  | P*cbh1*-250 qF | CCTTCGGCCTTTGGGTGTA |
|  | P*cbh1*-250 qR | TCTTTATCGGCTATTGTTCTTGG |
|  | P*cbh1*-TATA qF | TAGCCAAGAACAATAGCCGATAA |
|  | P*cbh1*-TATA qR | TTTCTGTGCCTCAAAAGATGGT |
|  | CBH1-ORF qF | CTTGGCAACGAGTTCTCTT |
|  | CBH1-ORF qR | TGTTGGTGGGATACTTGCT |
| *cbh2* | P*cbh2*-300 qF | TTTCTGCCCTTCCCACTAACT |
|  | P*cbh2*-300 qR | CCCAATAAAGAAGCTGGTTGAA |
|  | P*cbh2*-TATA qF | CCGGGCTTTATCCTGTGCTC |
|  | P*cbh2*-TATA qR | TACAAGCCTCTTCAGGTGAGC |
| *eg1* | P*eg1*-250 qF | AGTAGCATTGGAAACCGTAAA |
|  | P*eg1*-250 qR | TCTGTTAGGCTATGAGATGTTGG |
|  | P*eg1*-TATA qF | GGCATCTGTATCGCTCAAACT |
|  | P*eg1*-TATA qR | CTGATGGCGACAAGACAAAGT |
| *bgl1/cel3a* | P*bgl1*-850 qF | GGCAGGTCGATTCTCGGTAAGT |
|  | P*bgl1*-850 qR | GACAAGAAGCCAGCCGAGGG |
|  | P*bgl1*-TATA qF | GTTCCTTCAGTAACGCCACCCT |
|  | P*bgl1*-TATA qR | TGTTCGGTAACGCATTTCTGATT |
| *bgl2/cel1a* | P*bgl2*-500 qF | ATAGACCCTTCCCGTCCACCTG |
|  | P*bgl2*-500 qR | TGCTGATTGCGATAGACCCTGC |
|  | P*bgl2*-TATA qF | ACCAACGAACACGCTATTCTC |
|  | P*bgl2*-TATA qR | CAAACACGCAACTCCTCACTC |
| *actin1* | P*actin*-TATA qF | CATCGTGGCAGCGGAGTTA |
|  | P*actin*-TATA qR | TTGAAGAGGGCGAAGATAGACA |
