# Supplementary material for: Trichoderma reesei XYR1 activates cellulase gene expression via interaction with the Mediator subunit TrGAL11 to recruit RNA polymerase II
Source: PLoS Genet. 2020 Sep 2;16(9):e1008979. doi: 10.1371/journal.pgen.1008979 (PMC7467262; doi:10.1371/journal.pgen.1008979)
Supplement: S3 Table — (DOCX) [file pgen.1008979.s003.docx]

**S3 Table. *T. reesei* strains used in this research**

| **Strain name** | **Genotype and** **Characteristics** | **Reference** |
| --- | --- | --- |
| QM9414 | MAT1-2, cellulase higher producer derivative from the wild type strain QM6a |  |
| QM9414Δ*pyr4* | MAT1-2, Δ*pyr4*::*ptrA* | [1] |
| Δ*Trgal11* | MAT1-2, Δ*pyr4*::*ptrA*, Δ*gal11*::*pyr4* | This study |
| Δ*xyr1* | MAT1-2, Δ*xyr1*::*amds* | [2] |
| OE*xyr1* | MAT1-2, Δ*xyr1*::*amds*, P*tcu1*-*xyr1*::*hph* | [3] |
| OEX_ Δ*Trgal11* | MAT1-2, P*tcu1*-*xyr1*::*hph,* Δ*gal11*::*pyr4* | This study |
| OEX_TrGAL11-proA | MAT1-2, P*tcu1*-*xyr1*::*hph,* P*gal11*-*gal11*-*proA* | This study |
| Δ*Trmed3* | MAT1-2, Δ*pyr4*::*ptrA*, Δ*med3*::*pyr4* | This study |
| Δ*Trmed5* | MAT1-2, Δ*pyr4*::*ptrA*, Δ*med5*::*pyr4* | This study |
| Δ*Trmed16* | MAT1-2, Δ*pyr4*::*ptrA*, Δ*med16*::*pyr4* | This study |

**References**

1. Wang L, Zheng F, Zhang W, Zhong Y, Chen G, Meng X, et al. A copper-controlled RNA interference system for reversible silencing of target genes in *Trichoderma reesei*. Biotechnology for biofuels. 2018;11:33. Epub 2018/02/17. doi: 10.1186/s13068-018-1038-7. PubMed PMID: 29449881; PubMed Central PMCID: PMCPmc5806297.

2. Stricker AR, Grosstessner-Hain K, Wuerleitner E, Mach RL. Xyr1 (xylanase regulator 1) regulates both the hydrolytic enzyme system and D-xylose metabolism in *Hypocrea jecorina*. Eukaryotic cell. 2006;5(12):2128-37. doi: 10.1128/ec.00211-06. PubMed PMID: WOS:000243175500019.

3. Cao Y, Zheng F, Wang L, Zhao G, Chen G, Zhang W, et al. Rce1, a novel transcriptional repressor, regulates cellulase gene expression by antagonizing the transactivator Xyr1 in *Trichoderma reesei*. Molecular microbiology. 2017;105(1):65-83. doi: 10.1111/mmi.13685. PubMed PMID: 28378498.
