# Supplementary figures and images for: Trichoderma reesei XYR1 activates cellulase gene expression via interaction with the Mediator subunit TrGAL11 to recruit RNA polymerase II
Source: PLoS Genet. 2020 Sep 2;16(9):e1008979. doi: 10.1371/journal.pgen.1008979 (PMC7467262; doi:10.1371/journal.pgen.1008979)

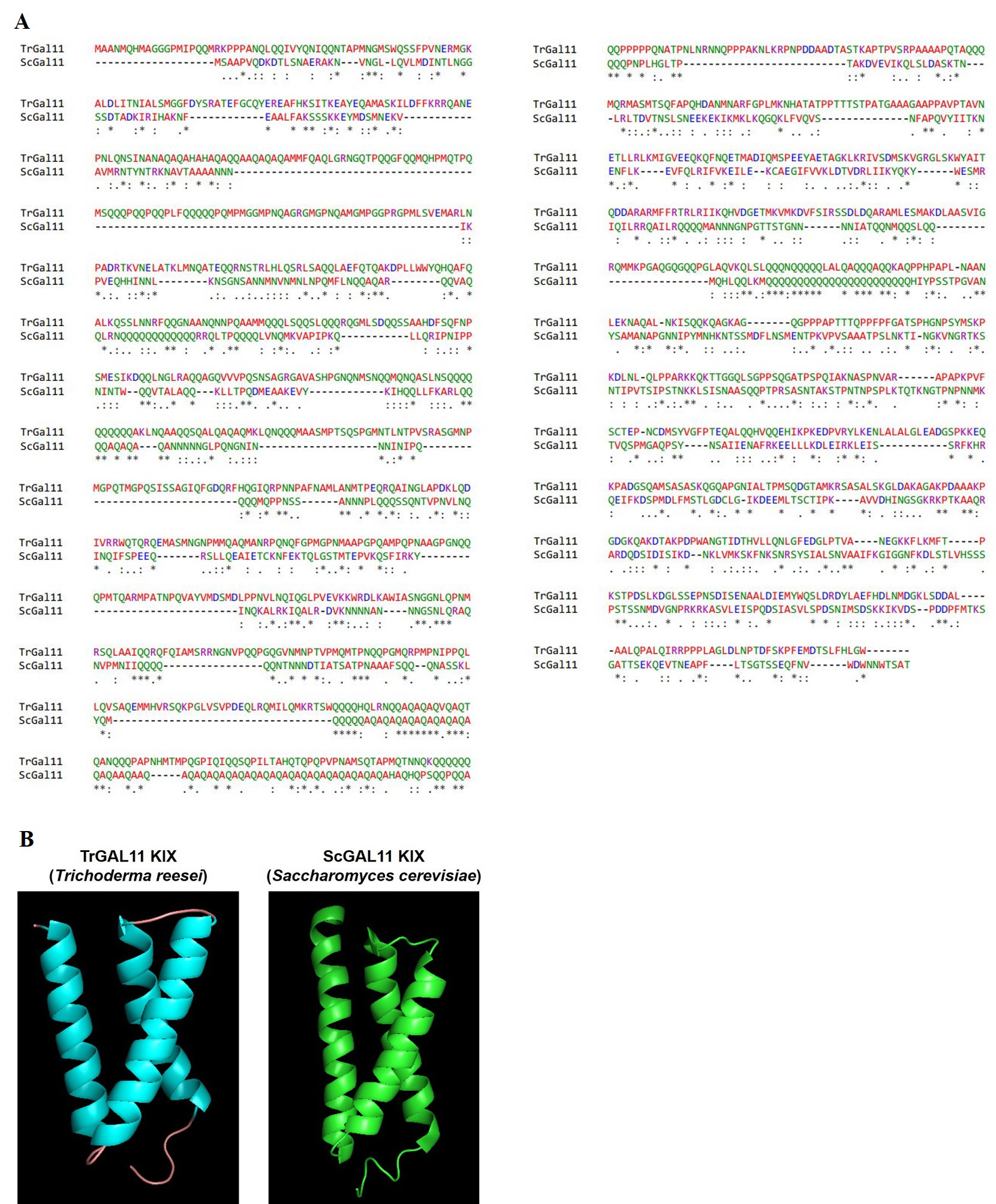

Supplement: S1 Fig — (A) Protein sequence alignment was performed by the Multiple Sequence Alignment tool MUSCLE (https://www.ebi.ac.uk/Tools/msa/muscle/) with the primary amino acid sequence of TrGAL11 and ScGAL11. (B) Secondary structural simulation of TrGAL11 KIX domain via SWISS-MODEL (https://swissmodel.expasy.org/) and structural comparison with ScGal11 KIX domain (PDB_2k0n). (TIF) [file pgen.1008979.s004.tif]

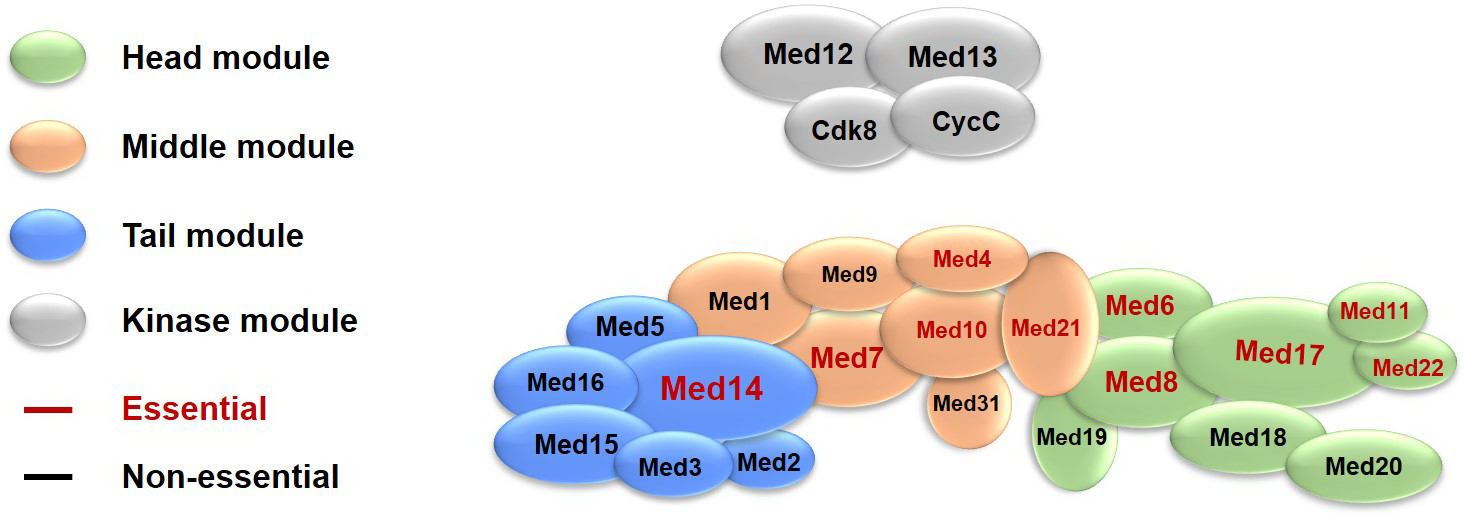

Supplement: S2 Fig — Compositional organization of the S. cerevisiae Mediator complex was modified from references [5, 61]. (TIF) [file pgen.1008979.s005.tif]

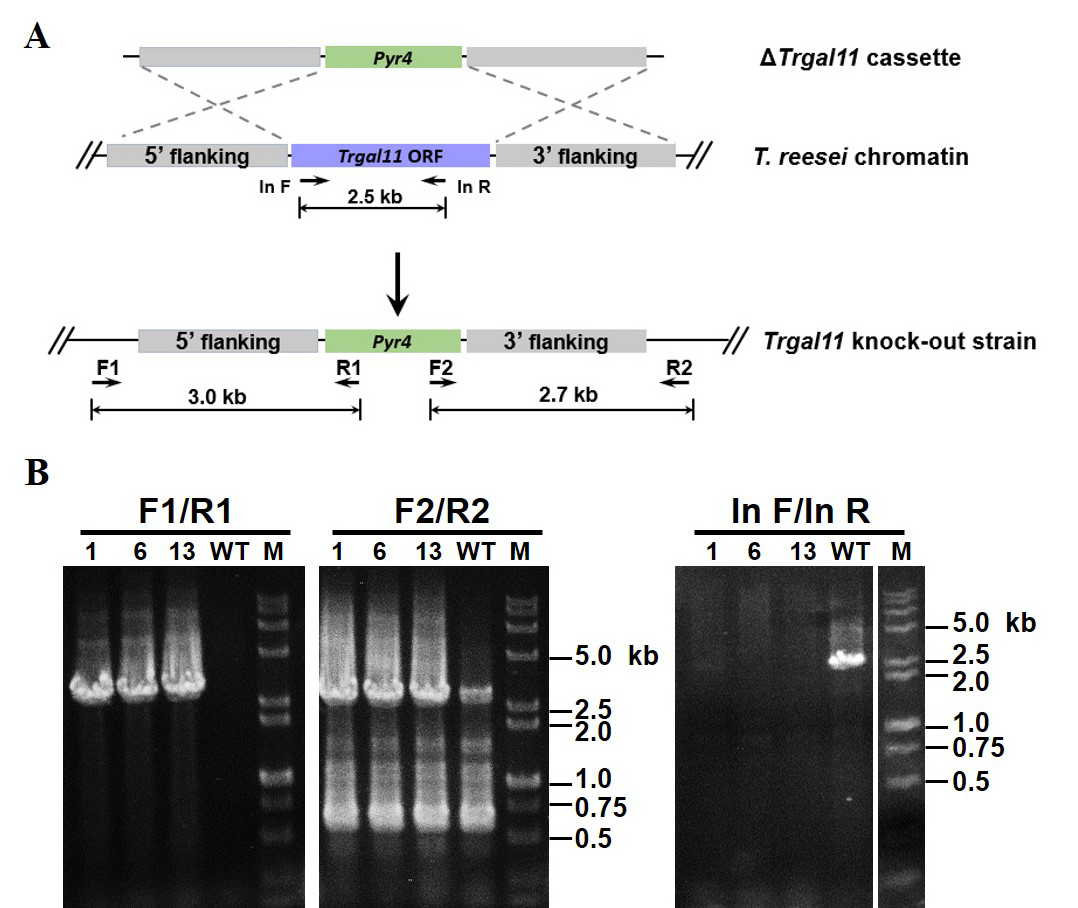

Supplement: S3 Fig — (A) Schematic illustration of the homologous integration of the T. reesei pyr4 gene at the Trgal11 locus resulting in the deletion of the coding sequences of Trgal11. (B) Diagnostic PCR was performed to verify the correct integration of the pyr4 gene at the Trgal11 locus. Lanes 1–3, the genomic DNA from three independent transformants was used as template; NC, the QM9414 genomic DNA was used as template. (TIF) [file pgen.1008979.s006.tif]

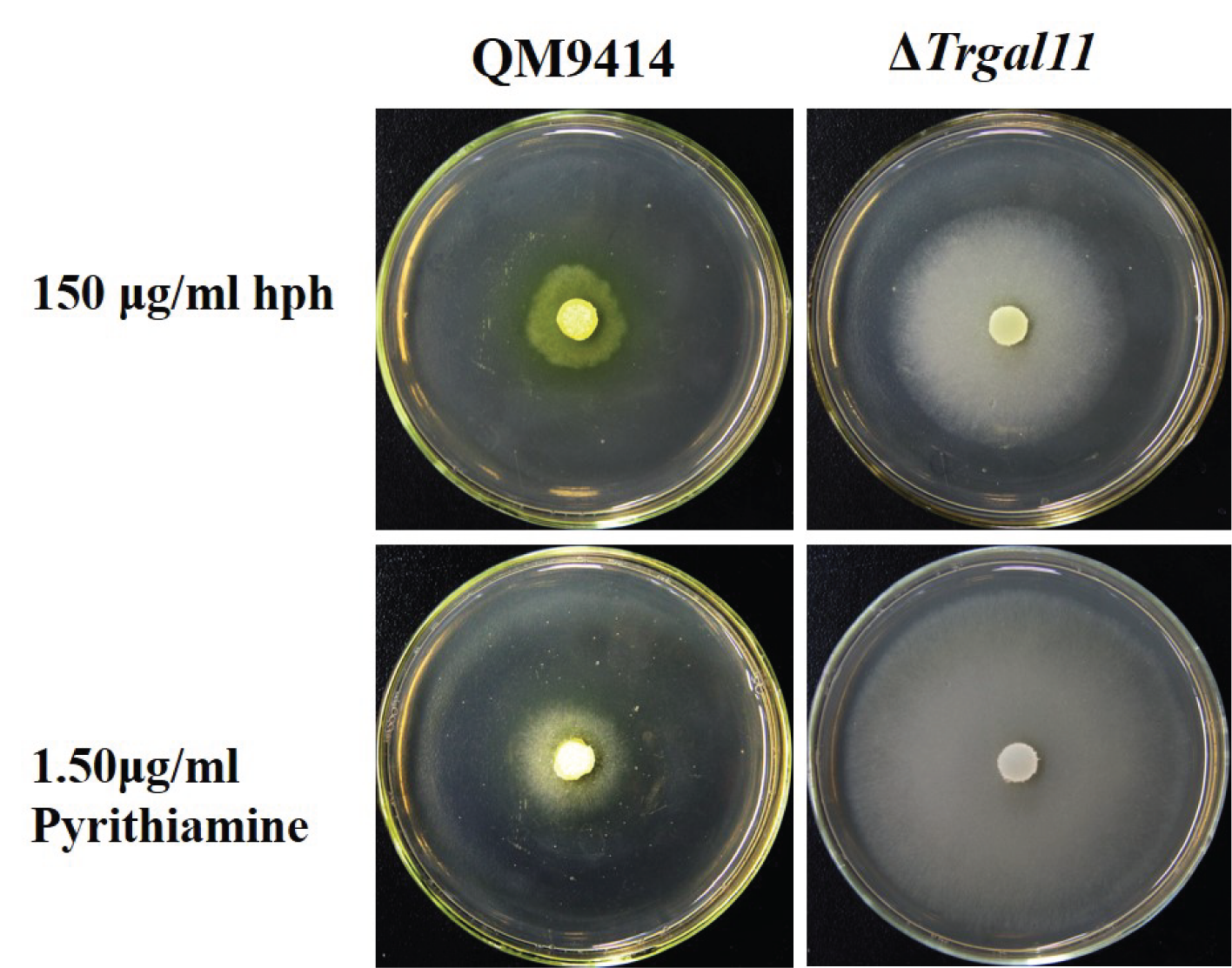

Supplement: S4 Fig — Growth of QM9414 and ΔTrgal11 strains on MM plates with hygromycin B or pyrithiamine. The result shown represented one of at least two independent experiments. (TIF) [file pgen.1008979.s007.tif]

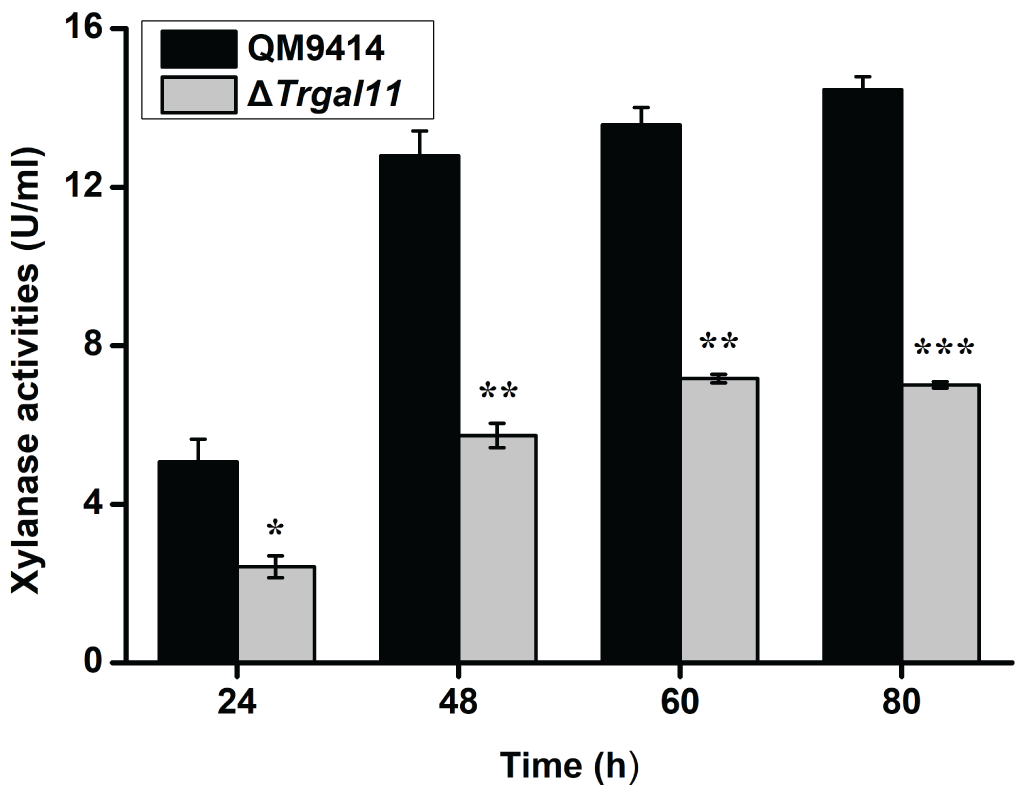

Supplement: S5 Fig — Xylanase activity of the supernatant from the parental strain QM9414 and ΔTrgal11 cultures on 1% (w/v) xylan for the indicated time periods. Significant differences (t-test, *P<0.05, **P<0.01, ***P<0.001) were detected for the extracellular activities between QM9414 and ΔTrgal11 for the indicated time points after induction. (TIF) [file pgen.1008979.s008.tif]

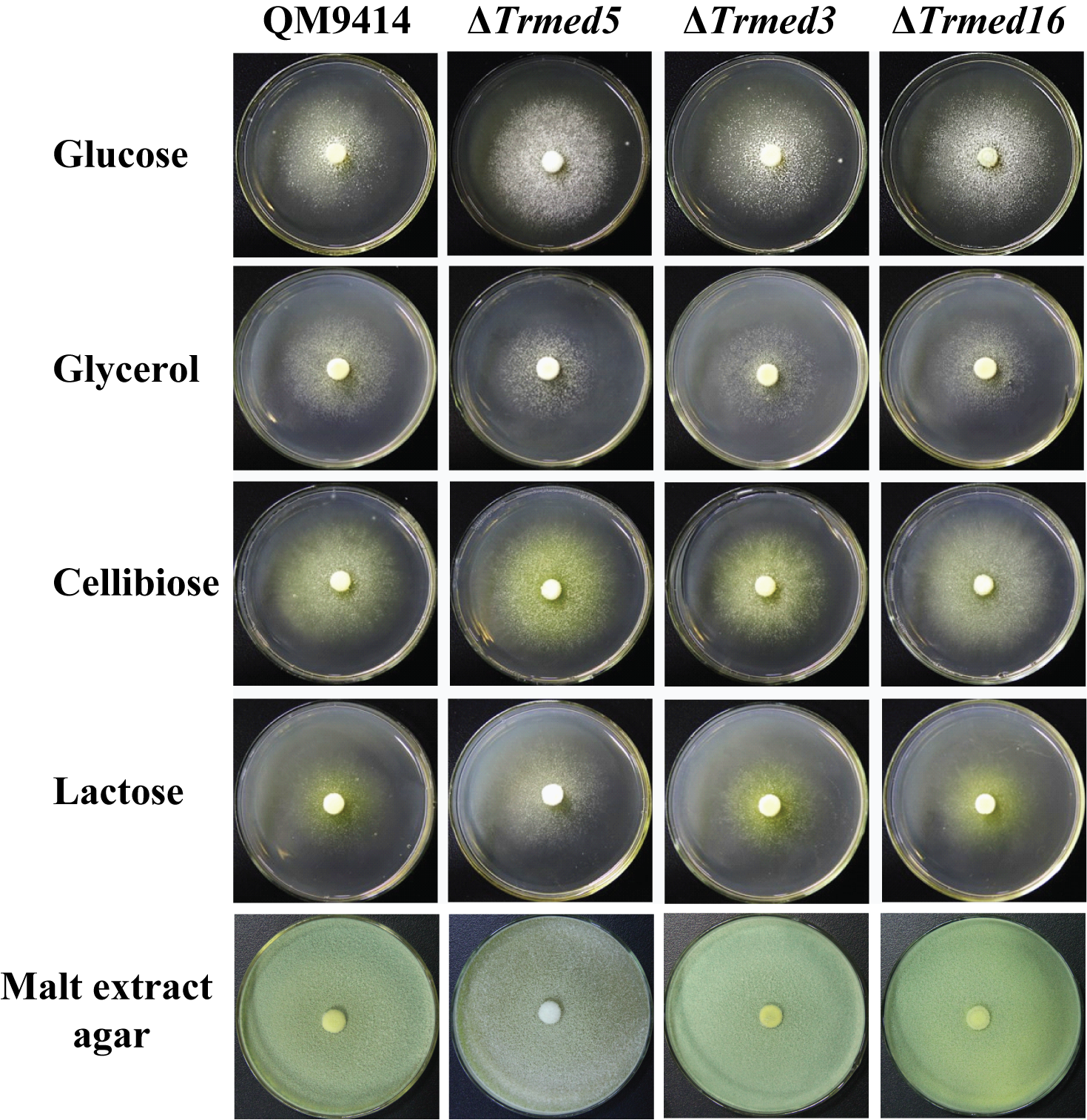

Supplement: S6 Fig — Growth and conidiation analysis of QM9414 and Trmed5, Trmed3, or Trmed16 deletion strains on plates with various carbon sources at a final concentration of 1% (w/v) at 30°C for 3 days or on malt extract agar for 5 days. Conidiation was neither significantly compromised except Trmed5 disruption. (TIF) [file pgen.1008979.s009.tif]

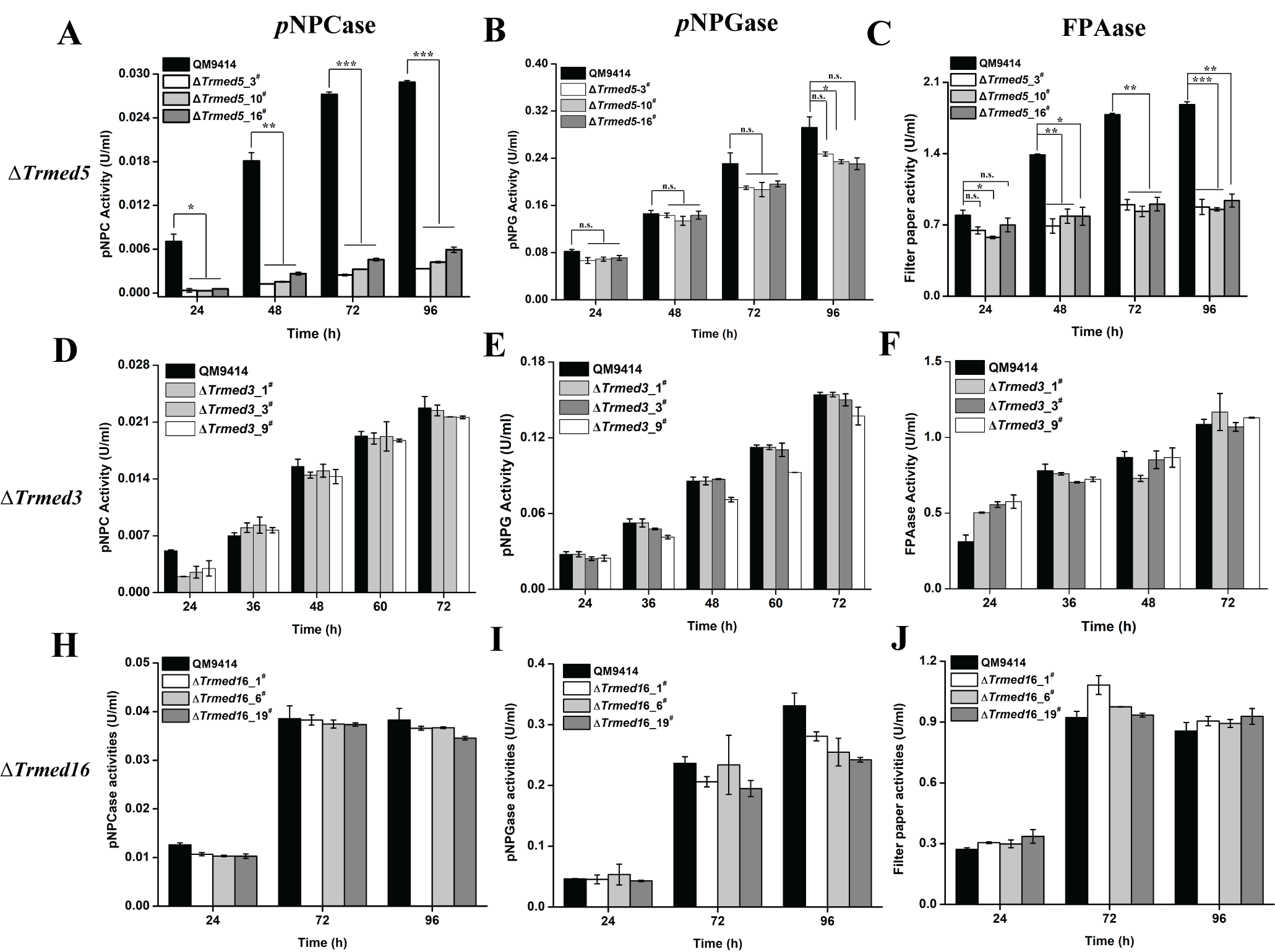

Supplement: S7 Fig — Extracellular pNPC (A, D, H), pNPG (B, E, I), and filter paper activities (FPA) (C, F, J) of the supernatant from the parental strain QM9414 and three independent transformants of ΔTrmed5, ΔTrmed3, or ΔTrmed16 cultured on 1% (w/v) Avicel were determined for the indicated time periods, respectively. (TIF) [file pgen.1008979.s010.tif]

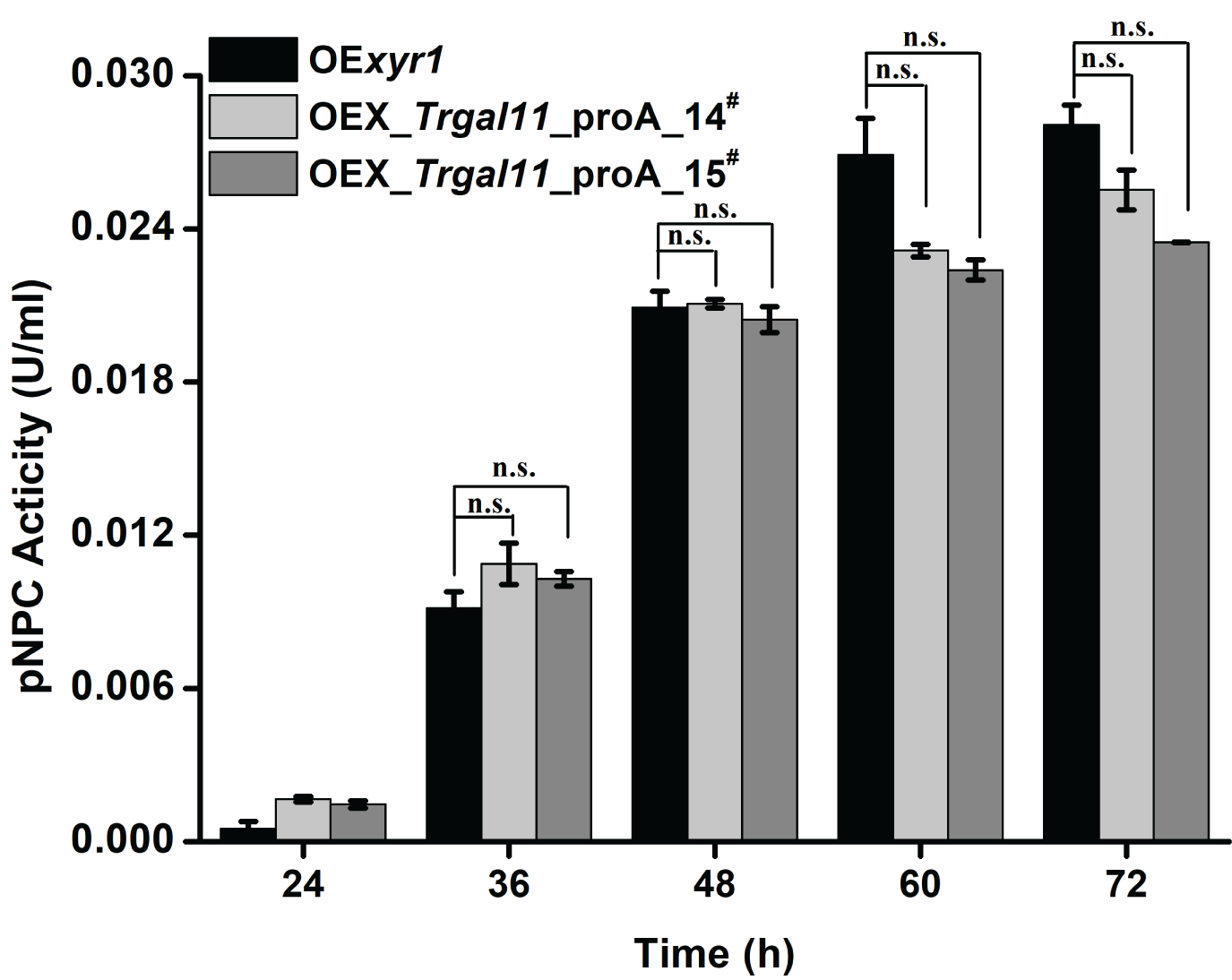

Supplement: S8 Fig — Extracellular pNPC activity of the supernatant from OExyr1 and two independent OEX_Trgal11-proA transformants cultured on 1% (w/v) Avicel for the indicated time periods. No significant differences (t-test, P>0.05, n.s.) were detected for the pNPC activities between OExyr1 and two independent OEX_Trgal11-proA transformants under induction condition. (TIF) [file pgen.1008979.s011.tif]

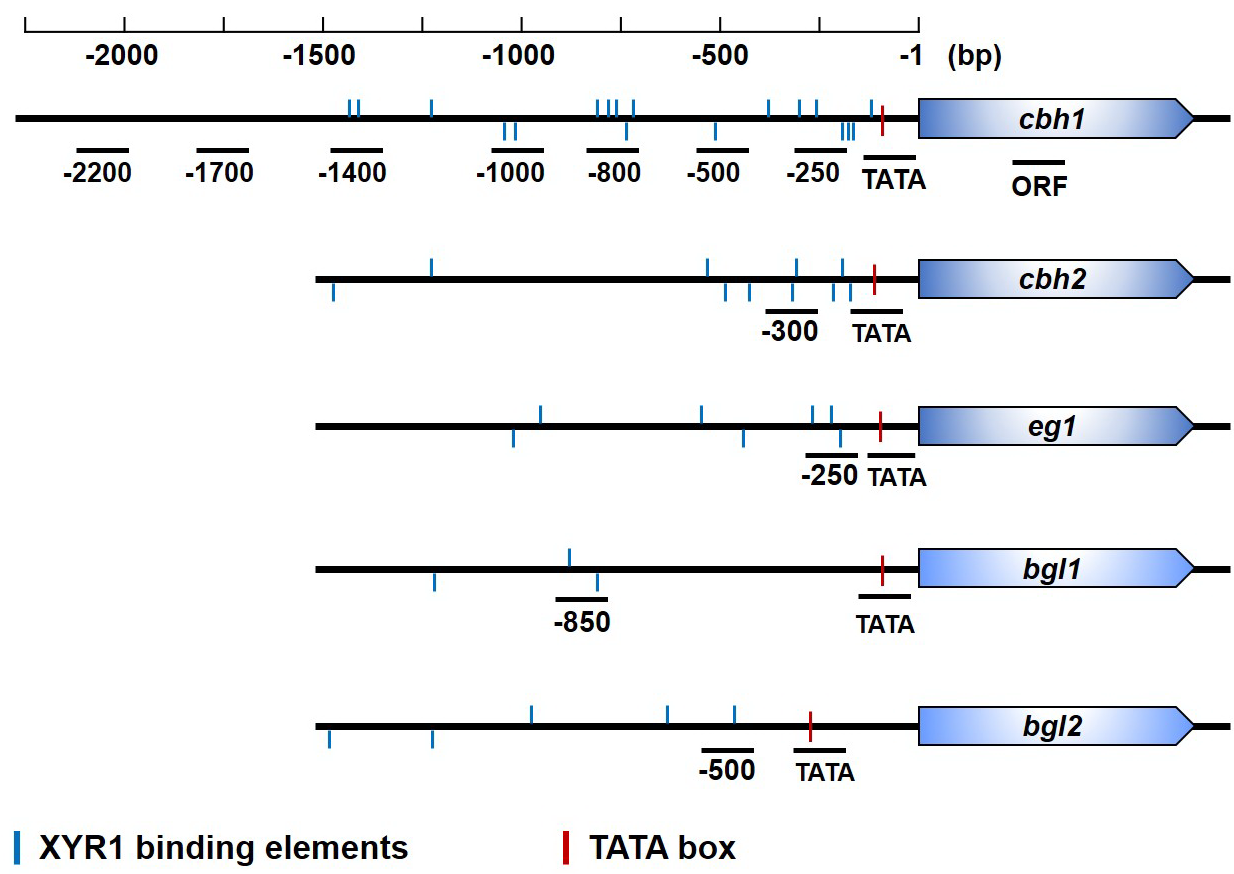

Supplement: S9 Fig — The number below each short bar denotes the approximate position of the amplified promoter regions relative to the start codon ATG. XYR1 binding elements GGC(T/A)3 and the TATA box were labeled as vertical bars [44]. (TIF) [file pgen.1008979.s012.tif]
